# Engineered Interpenetrating MXene Networks in Aramid Layered Films for Antioxidant and Broadband Electromagnetic Interference Shielding

**DOI:** 10.1002/advs.202520793

**Published:** 2026-01-20

**Authors:** Hongli Cheng, Gaojie Han, Congqi Liu, Yang Zhou, Yiming Wang, Bing Zhou, Yuezhan Feng, Chuntai Liu, Hao‐Bin Zhang, Changyu Shen

**Affiliations:** ^1^ State Key Laboratory of Structural Analysis Optimization and CAE Software for Industrial Equipment National Engineering Research Center for Advanced Polymer Processing Technology Zhengzhou University Zhengzhou China; ^2^ State Key Laboratory of Organic‐Inorganic Composites College of Materials Science and Engineering Beijing University of Chemical Technology Beijing China

**Keywords:** ANF/MXene film, Antioxidant, EMI shielding, interpenetrating layered structure

## Abstract

MXene‐based layered films featuring a “brick‐and‐mortar” architecture show significant potential for electromagnetic interference (EMI) shielding applications; however, the insulating polymer “mortar” disrupts the connectivity of the MXene network, ultimately compromising both electrical conductivity and shielding effectiveness (SE). To address this limitation, this study introduces an innovative interpenetrating layered structure, comprising independent aramid nanofiber (ANF) layers and MXene layers, fabricated through a directional freeze–thaw intercalation–gel‐film formation strategy. Unlike traditional homogeneous layered films, this unique layered structure features mutually embedded conductive and insulating layers, facilitating efficient electron transport, generating numerous heterogeneous interfaces for multiple reflections and scattering, and enhancing oxidation resistance. The interpenetrating ANF/MXene film exhibits exceptional electrical conductivity (5630.8 S/m) and EMI SE (43.3 dB) at ∼40 wt.% MXene loading, significantly outperforming the homogeneous ANF/MXene film (26.9 dB). Importantly, the interpenetrating ANF layers fully encapsulate the MXene layers, providing remarkable long‐term stability with only a 10% decline in EMI performance after 80 days of aging. Furthermore, the interpenetrating ANF layers form a resilient mechanical framework, resulting in the A@M film boasting outstanding mechanical properties (tensile strength of 121.0 MPa, fracture strain of 13%). Consequently, this work presents a novel design and approach for fabricating high‐performance MXene‐based layered EMI shielding films.

## Graphical abstract

This study introduces an innovative interpenetrating layered structure, comprising alternating aramid nanofiber (ANF) layers and MXene layers, fabricated through a directional freeze–thaw intercalation–gel‐film formation strategy. Unlike traditional homogeneous layered films, this unique layered structure features mutually embedded conductive and insulating layers, facilitating efficient electron transport, generating numerous heterogeneous interfaces for multiple reflections and scattering, and enhancing oxidation resistance.

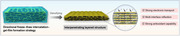

## 1 Introduction

With the increasing awareness of electromagnetic wave (EMW) radiation and the complexities of electromagnetic environments, the issue of EMW pollution has become a critical concern. Electromagnetic interference (EMI) shielding represents one of the simplest techniques to control EMW pollution [1, 2, 3, 4]. However, conventional EMI shielding materials, such as metals and ceramics, often fail to meet the next‐generation requirements for “high‐performance, broadband, lightweight, and thin” solutions. In contrast, conductive polymer composites have emerged as promising candidates for next‐generation EMI shielding materials owing to their lightweight, cost‐effectiveness, flexibility, processability, and corrosion resistance [5, 6, 7, 8, 9].

**SCHEME 1:**
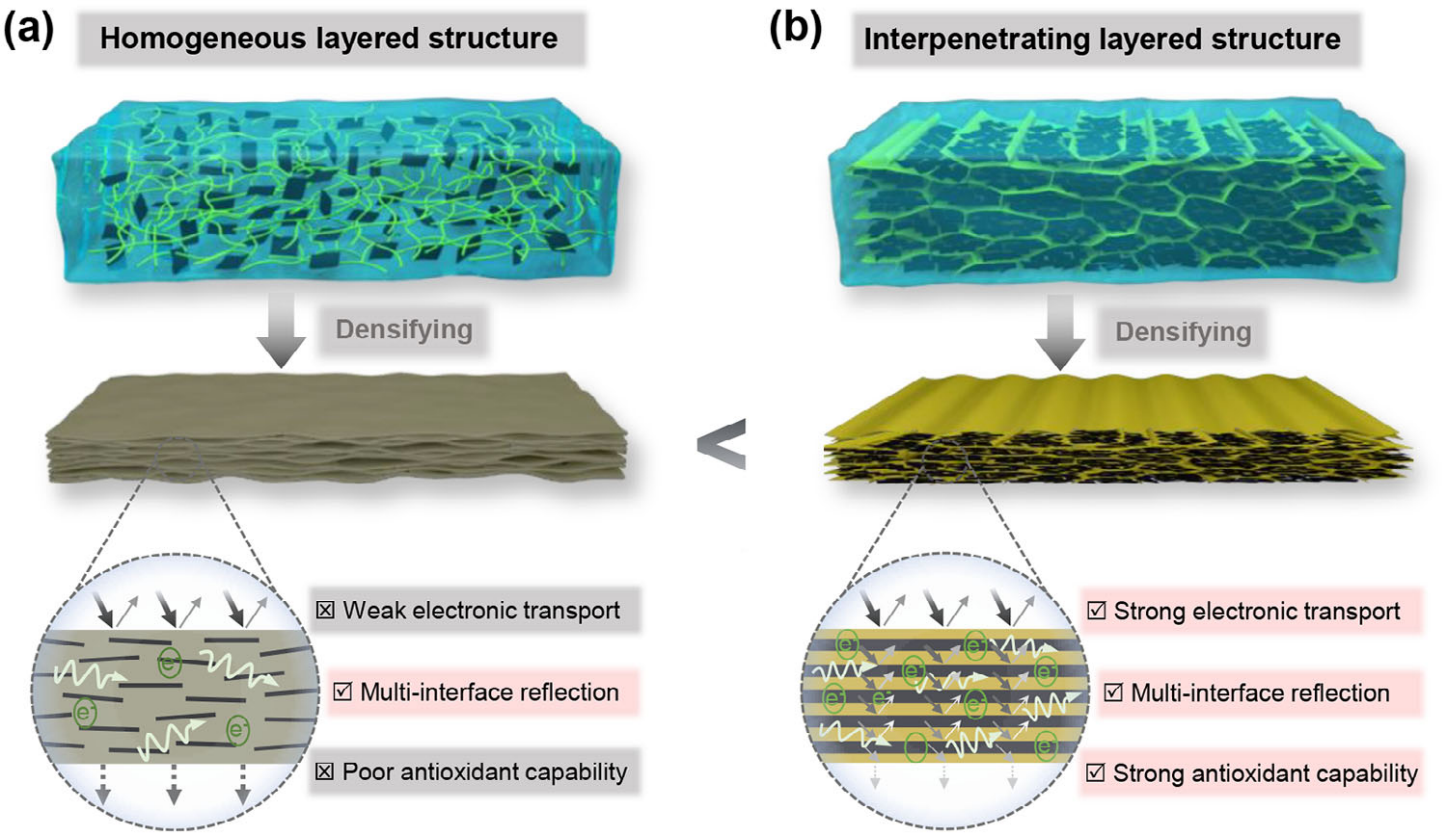
Illustrations for preparations, structures, EMI shielding mechanisms, advantages, and disadvantages of (a) traditional homogeneous layered film and (b) interpenetrating layered film.

The biomimetic layered film, featuring a “brick‐to‐mortar” structure, exhibits significant advantages over conventional block‐type EMI shielding materials [10, 11, 12, 13]. Primarily, it can achieve an optimal shielding effect at an ultra‐thin thickness (*d*<100 µm), meeting the critical requirements of lightweight design and miniaturization. Additionally, the highly aligned one‐ or two‐dimensional (1D/2D) conductive fillers, in conjunction with the dense “brick‐to‐mortar” architecture, endow the layered film with remarkable mechanical strength, toughness, and foldable flexibility, making it suitable for a wide range of real‐world applications [14, 15, 16, 17]. Importantly, its versatile preparation techniques—such as vacuum‐assisted filtration and evaporation self‐assembly—demonstrate substantial potential for large‐scale production [18, 19, 20, 21, 22, 23, 24]. Nonetheless, the ultra‐thin nature of layered structural EMI shielding films poses considerable challenges. According to Schelkunoff's formulas [25],

(1) $$SE_{R}=168.2+10\;\mathrm{lo}g_{10}\left(\frac{\sigma_{r}}{f\mu_{r}}\right)$$

(2) $$SE_{A}=131.43d\;{\left(f\sigma_{r}\mu_{r}\right)}^{1/2}$$

where *f* denotes the frequency, while *d, σ_r_*, and *µ_r_* are the thickness and relative conductivity, and relative permeability of shielding materials, respectively. The ultra‐thin layered structure beneath the skin depth necessitates high conductivity (*σ* >100 S/m) to facilitate effective EMI shielding through robust reflection (SE_R_) and absorption (SE_A_) losses [26, 27]. However, as illustrated in Scheme 1a, in traditional homogeneous layered structures, the insulating polymer “mortar” blocks the direct interconnection of conductive fillers, thereby constraining both electrical conductivity and EMI shielding performance [28, 29, 30, 31]. While the abundant heterogeneous interfaces within homogeneous layered films can induce multiple reflection‐scattering, their EMI shielding capabilities remain limited, often requiring a high content of conductive fillers (>50 wt.%) for substantial shielding effectiveness [32, 33]. Additionally, high concentrations of conductive fillers without protective polymer encapsulation are inherently prone to oxidation.

In recent years, 2D MXenes have emerged as an advanced class of materials, demonstrating metal‐like electrical conductivity and intrinsic self‐assembly attributes that facilitate the formation of freestanding conductive layers within layered structural films [20, 34, 35, 36, 37]. This independent conductive layer configuration effectively reduces interlayer contact resistance, while the supporting polymer layer sustains the film's structural integrity and mechanical properties. Consequently, innovative multilayer structural designs—such as Janus [38, 39], sandwich‐like [40, 41], and alternating layered configurations [42]—have rapidly gained traction, spurring new research hotspots and breakthroughs in the development of high‐performance layered EMI shielding films. Our team successfully fabricated a freestanding composite film featuring an alternating layer structure composed of cellulose nanofiber (CNF) and MXene layers via an alternating filtration process [34]. The freestanding MXene layers effectively mitigate the insulating effects posed by the polymer matrix, resulting in a significant enhancement of both conductivity and EMI shielding performance in the alternating layered film. Notably, this optimized layered structure achieved a high shielding effectiveness (SE, ∼40 dB) with a relatively low MXene content of 50 wt.%. However, due to preparative constraints, this alternating multilayer structure lacks sufficient heterogeneous interfaces, which weakens the internal multiple reflection‐scattering effect. Moreover, the freestanding MXene layers are vulnerable to oxidation in the absence of polymer encapsulation.

Compared to the simple alternating multilayer structure, further subdividing the distribution of independent MXene layers to form a continuously interpenetrating layered structure comprising thousands of MXene layers (Scheme 1b), not only facilitates efficient and unobstructed electron transport within individual MXene layers but also generates numerous heterogeneous interfaces that promote multiple reflections and scattering of EMWs. This synergy leads to highly effective EMI shielding performance, even with a relatively low MXene content of less than 50 wt.%. Moreover, the interactive polymer layer also acts as a robust barrier, protecting MXene from exposure to air and significantly improving its antioxidant properties. Nevertheless, the fabrication techniques for these interpenetrating layered structures continue to present considerable challenges.

In this work, we proposed a novel directional freeze–thaw intercalation–gel‐film formation strategy aimed at constructing an interpenetrating layered structure featuring mutually embedded MXene conductive layers and aramid nanofiber (ANF) layers. The interpenetrating layered structure overcomes the key limitations of traditional homogeneous films—poor conductivity, weak oxidation resistance, and insufficient mechanical robustness—through three synergistic design principles: (1) Continuous conductive network: Unlike traditional films where insulating polymer disrupts MXene connectivity, the interpenetrating structure forms uninterrupted MXene conductive layers within the ANF matrix, minimizing contact resistance and enabling exceptional electrical conductivity (5630.8 S/m) and impressive EMI SE of 43.3 dB at a relatively low MXene content of only 41 wt.%. (2) Mechanical reinforcement: The high‐strength ANF layers serve as the primary load‐bearing scaffold, allowing the A@M film to achieve a tensile strength of 121.0 MPa and a fracture strain of 13%. (3) Encapsulation protection: The ANF layers fully encapsulate MXene networks, creating a physical barrier against oxygen and moisture, thereby preventing oxidation and EMI shielding degradation with only a 10% reduction in EMI SE after 80 days. Therefore, this interpenetrating layered film offers considerable potential for practical applications in advanced EMI shielding.

## 2 Results and Discussion

### 2.1 Fabrication, Morphology, and Structure of Interpenetrating ANF/MXene Film

As is well known, in the traditional gel densification process, the organic “mortar” and inorganic “brick” components are uniformly deposited and assembled, resulting in the homogeneous layered structural films (Scheme 1a). These homogeneous layered films typically suffer from poor electron transport due to the insulation of conductive fillers within the polymer matrix, which fundamentally limits their EMI shielding effectiveness. To conquer this limitation, herein, we introduce a novel interpenetrating layered structure that features mutually and continuously embedded conductive and matrix layers (Scheme 1b). This innovative structure not only facilitates efficient electron transport within the interpenetrating conductive layers but also generates abundant heterogeneous interfaces for enhanced reflection and scattering. Additionally, the interpenetrating matrix layers protect the conductive layers against air exposure, significantly improving their oxidation resistance. To achieve the interpenetrating layered structure, a novel directional freeze–thaw intercalation–gel‐film formation strategy was developed in this study.

**FIGURE 1:**
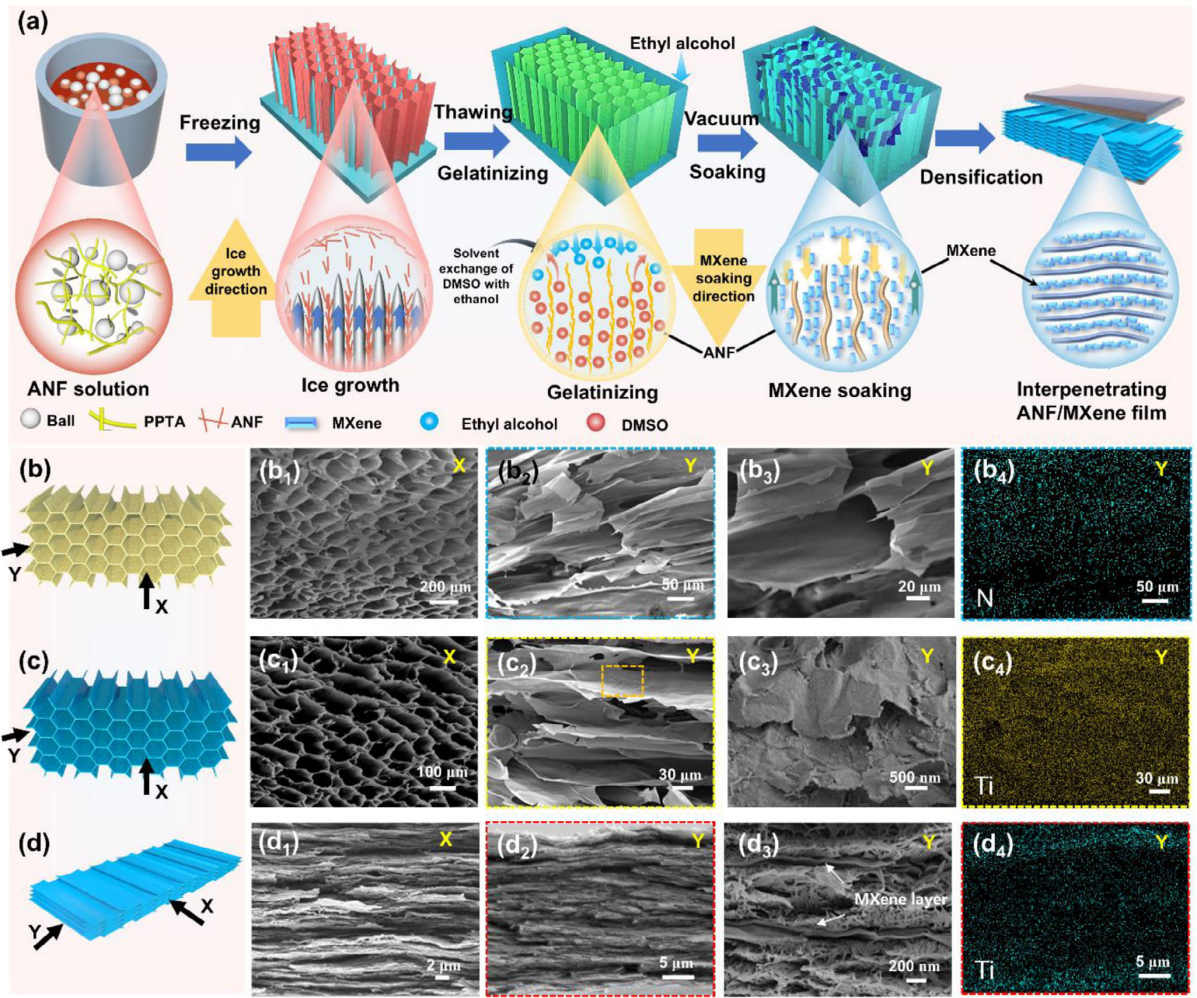
Preparation, morphology, and structure evolution of interpenetrating ANF/MXene layered films: (a) Schematic illustrating the fabrication process of A@M_x_ films. Schematic diagram, cross‐sectional SEM images with different magnifications and EDS mappings of (b) thawing ANF hydrogel, (c) soaking ANF/MXene hydrogel, and (d) A@M_x_ films.

Figure 1a illustrates the fabrication process of the interpenetrating ANF/MXene (A@M) layered films. Typically, the ANF solution underwent conventional unidirectional freeze casting to create 3D interconnected honeycomb skeletons. This process was facilitated by the exclusion effect of unidirectionally growing DMSO crystals (Figure S1). Following this, the frozen ANF blocks were immersed in a pre‐cooled (−20°C) ethanol solution for thawing and gelation. During this stage, the simultaneous processes of solvent exchange and in situ protonation effectively preserved the 3D honeycomb architecture templated by the ice growth in the resulting ANF hydrogel. Subsequently, the ANF honeycomb hydrogel was soaked in MXene aqueous solution. The combined effects of the diffusion driven by MXene concentration gradients and the Marangoni effect among H_2_O/ethanol solvents enabled the precise and controllable assembly of MXene nanosheets into the honeycomb pores within the ANF hydrogel (Figure S2). Finally, the obtained ANF/MXene hydrogels underwent slow hot‐pressing in a direction perpendicular to the ice crystal growth, effectively expelling excess water during drying and culminating in the formation of dense A@M films (Figure S3). Throughout the densification process, the honeycomb‐like pore walls and pore fillers were assembled into layers of ANF matrix and MXene conductive layers, resulting in an interpenetrating yet continuous layered structure. The dense honeycomb microstructure formed by ice crystal growth leads to A@M films with over 50 interpenetrating MXene and ANF layers. Additionally, the content of MXene in A@M films can be flexibly adjusted by varying the concentration of the soaking MXene solution. For comparison, a homogeneous ANF/MXene layered film (AM) was fabricated via vacuum‐assisted filtration.

The interpenetrating layered structure essentially relies on the effective infiltration of MXene nanosheets into the pores of the ANF honeycomb gel. Importantly, the pore size of the ANF gel—formed by unidirectional ice crystal growth through processes of repulsion and aggregation—plays a critical role in determining the infiltration of MXene nanosheets. As illustrated in Figure 1b and Figure S4, cross‐sectional scanning electron microscopy (SEM) analysis reveals that the ANF gel possesses a highly organized honeycomb pore architecture aligned along the X‐axis direction, characterized by uniform pore sizes and smooth internal walls. The pore size distribution spans from 100 to 200 µm, which is notably larger than the dimensions of the MXene nanosheets (1–10 µm), thereby facilitating their effective infiltration during the soaking process. As anticipated, the cross‐sectional SEM images (Figure 1c_1_; Figure S5) illustrate the successful interpenetration of MXene nanosheets into the ANF gel's pores while maintaining the integrity of the honeycomb structure. High‐magnification SEM images (Figure 1c_2_,c_3_), accompanied by elemental mappings of C, N, F, and Ti (Figure 1c_4_; Figure S6), further corroborate the homogeneous distribution of MXene nanosheets across the pore walls. Following pressure‐assisted densification, the ANF pore walls and embedded MXene nanosheets were densely stacked, collectively establishing an interpenetrating layered structure (Figure 1d; Figure S7). Additionally, high‐magnification SEM observations (Figure 1d_3_; Figure S7) validate that the MXene and ANF layers interpenetrate mutually. This interpenetrating configuration enables the MXene layers to form a continuous conductive network while being completely encapsulated by the ANF layers, significantly enhancing the material's antioxidant properties. In contrast, both the pure ANF film and vacuum‐filtered AM film exhibit typical homogeneous layered structures (Figures S8 and S9), where the insulating ANF matrix interspersed among the MXene nanosheets impedes efficient electron transport.

**FIGURE 2:**
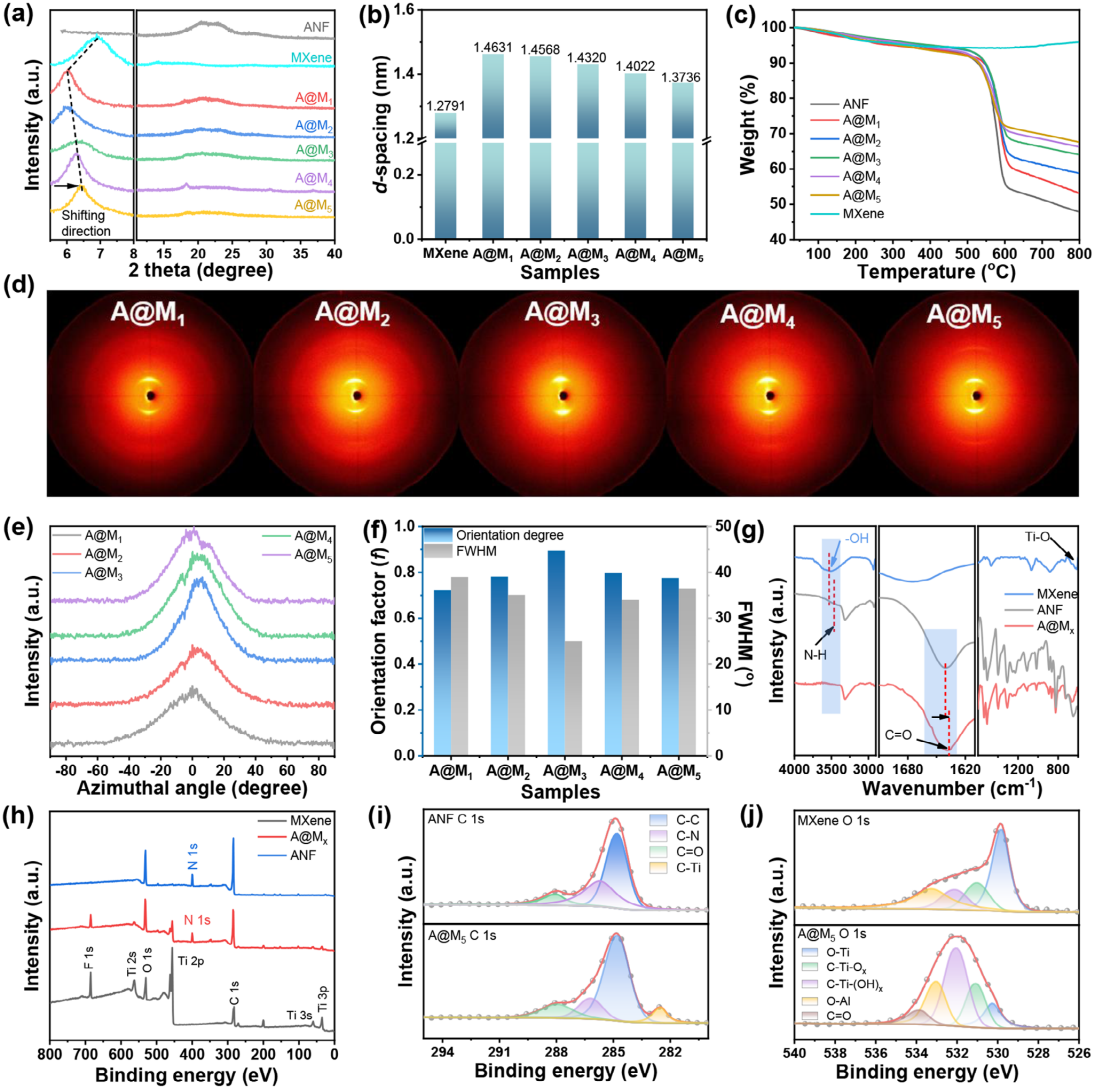
Physical and chemical structures of interpenetrating ANF/MXene layered films: (a) XRD patterns and (b) d‐spacing, (c) TGA curves of ANF, MXene, and A@M_x_ films. (d) 2D WAXD patterns, (e) azimuthal scan profiles of the MXene's (002) peak, and (f) orientation factor and FWHM of A@M_x_ films. (g) FTIR spectra, (h) XPS full spectra, high‐resolution XPS spectra at (i) C 1s and (j) O 1s, for ANF, MXene, and A@M_5_ film.

The interpenetrating layered structure is further substantiated by X‐ray diffraction (XRD) analysis. As depicted in Figure 2a, a noteworthy observation is the gradual shift of the (002) peak of MXene in the A@M_x_ films from 2θ = 6.91° to 6.04° with increasing MXene content. Utilizing the Bragg equation, the *d*‐spacing of MXene was calculated and illustrated in Figure 2b. The results demonstrate an increase in *d*‐spacing from 1.2791 to 1.4631 nm, which clearly indicates the interpenetration of ANF layers between MXene layers, ultimately leading to the formation of the interpenetrated layered structure. Additionally, as the MXene immersion concentration escalated, the augmentation of MXene content within the pores led to denser film formation under constant pressure, resulting in a gradual decrease in the interlayer distance from 1.4631 to 1.3736 nm. Thermogravimetric analyses (TGA, Figure 2c) further corroborated these findings, showing that the MXene content and density increased from 10.9 to 41.1 wt.% and from 1.036 to 1.248 g cm^−3^ as the concentration of the MXene immersion solution was elevated (Table S1). Moreover, the TGA results indicated that the A@M_x_ films exhibit exceptional thermal stability, with a decomposition temperature ranging from 500–600°C (Figure S10), demonstrating their high‐temperature application potential.

The ordered honeycomb structure formed via unidirectional freezing enhances the establishment of a well‐aligned conductive network, the degree of orientation of which can be quantitatively assessed using wide‐angle X‐ray diffraction (WAXD). All A@M_x_ films display anisotropic 2D scattering patterns featuring a distinct polar arc in the (002) plane of MXene (Figure 2d). Correspondingly, the azimuthal profiles exhibit sharp anisotropic peaks with full width at half maxima (FWHM) ranging from 25° to 39° (Figure 2e,f). These observations confirm a highly oriented distribution of MXene within the A@M_x_ films. Furthermore, Herman's orientation functions (Equations (3) and (4)) were employed to evaluate the orientation factor (*f*) [43, 44, 45]:

(3) $$f=\frac{3\langle \cos^{2}\theta \rangle -1}{2}$$

(4) $$\langle \cos^{2}\theta \rangle =\frac{\int_{0}^{\frac{\pi }{2}}I\left(\theta \right)\cos^{2}\theta \sin\theta d\theta }{\int_{0}^{\frac{\pi }{2}}I\left(\theta \right)\sin\theta d\theta }$$

where 〈*cos* ^2^θ〉 denotes the average value of the squared azimuthal cosine for the (002) peak, and *I(θ)* represents the signal intensity at an azimuthal angle of *θ*. As illustrated in Figure 2f, the calculated *f* exhibits an initial increase followed by a decline as the concentration of MXene rises, attaining a peak value of 0.895 for the A@M_3_ film. The obtained results indicate that the interpenetrating MXene nanosheets demonstrate a high degree of in‐plane orientation, with *f* values spanning from 0.722 to 0.895. This well‐aligned conductive network significantly enhances the mechanical properties, electrical conductivity, and EMI shielding performance of the film.

Figure 2g and Figure S11 display the Fourier transform infrared spectra (FTIR) of MXene, ANF, and A@M film, highlighting characteristic absorption peaks at 3517, 628 cm^−1^ and 3326, 1641 cm^−1^, which correspond to the ─OH and Ti─O stretching vibrations of MXene and the N─H and C═O stretching vibrations of ANF, respectively [46, 47]. When compared to the individual ANF and MXene, the C═O and ─OH absorption peaks of the A@M film exhibit noticeable shifts from 1641 to 1637 cm^−^^1^ and from 3531 to 3467 cm^−^^1^, respectively. This observation suggests the formation of hydrogen bonds between ANF and MXene within the A@M film. Furthermore, the X‐ray photoelectron spectroscopy (XPS) survey reveals the presence of C, O, Ti, and F elements in the A@M film (Figure 2h). A comparison with the high‐resolution XPS spectra at C 1s and O 1s of ANF, MXene, and A@M film (Figure 2i,j) showed the presence of typical C─Ti (282.5 eV), C═O (288.0 eV), C─N (286.2 eV), O─Ti (530.3 eV), and C─Ti─Ox (531.1 eV) bonds in the A@M film. Notably, there is a significant increase in the peak areas of C═O and C─Ti─O_x_, while the peak area of Ti─O demonstrates a corresponding lessening. These alterations indicate notable modifications in the electronic environments of the C and O atoms in the A@M film undergo notable modifications compared to those in ANF and MXene, confirming the formation of hydrogen bonds between the C═O and ─OH groups. Consequently, these strong interlayer interactions enhance the interlocking effect of the interpenetrating layered structure, thereby further improving the mechanical properties of A@M films.

### 2.2 Mechanical Properties of the Interpenetrating ANF/MXene Film

**FIGURE 3:**
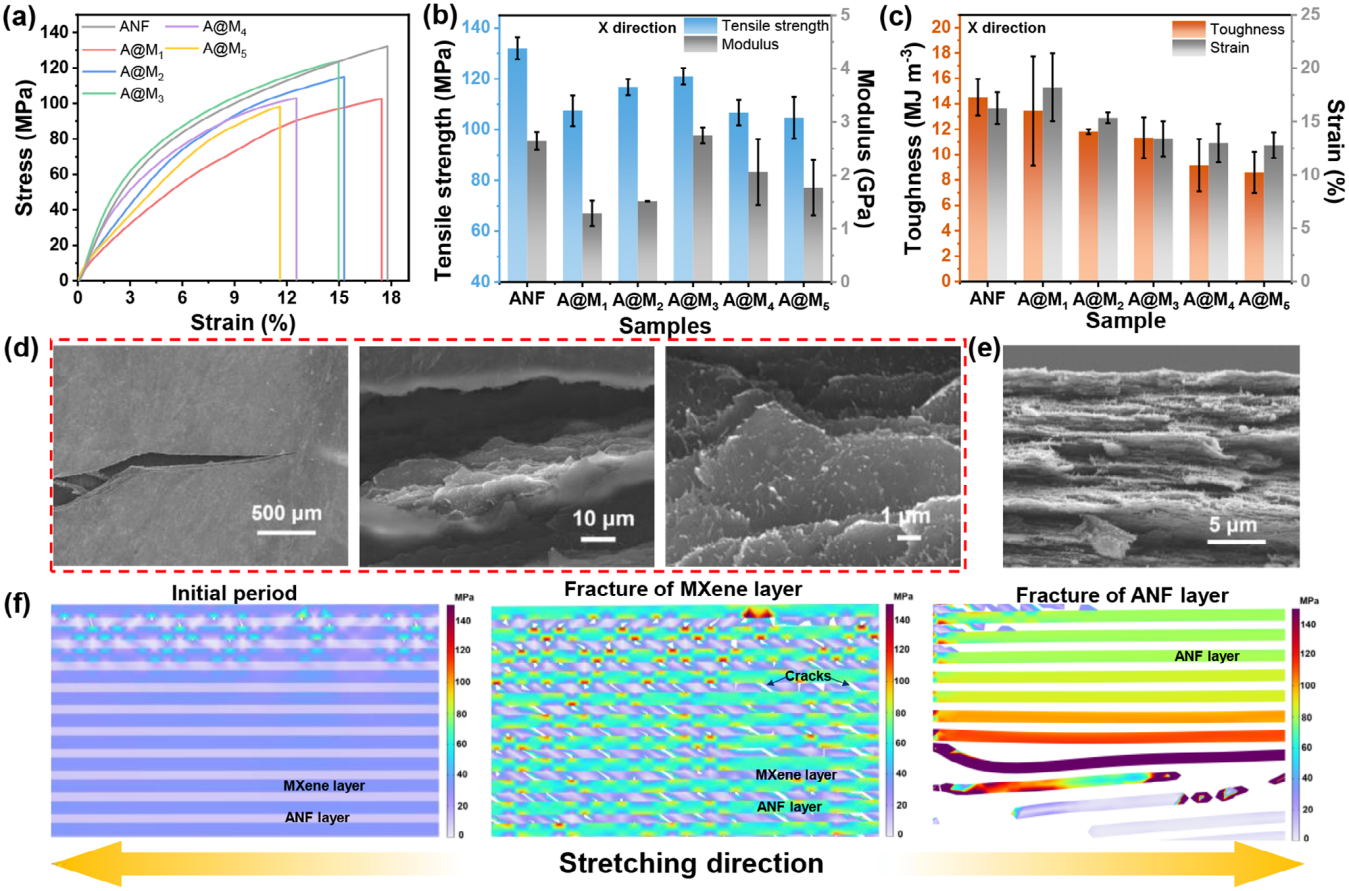
Mechanical properties of interpenetrating ANF/MXene layered films: (a) Stress–strain curves, (b) tensile strength and modulus, (c) toughness and fracture strain of pure ANF and A@M_x_ films. SEM images of (d) crack with different magnification and (e) tensile fracture surface for A@M_5_ film. (f) Finite element simulations of stress distribution for A@M_x_ film at different stretching stages.

The interpenetrating ANF layers form a robust mechanical support framework, thereby ensuring the structural integrity of A@M_x_ films. Concurrently, the aligned ANF network introduces a degree of anisotropy in the mechanical properties [34, 48]. Figure 3a–c and Figure S12 present the tensile properties of both pure ANF and A@M_x_ films, measured along the ice crystal growth direction (X‐direction) and its perpendicular direction (Y‐direction), respectively. In both the X‐ and Y‐directions, the mechanical properties of the A@M_x_ films are inferior to those of the pure ANF film, attributed to the inherently weak mechanical performance of the MXene layer. Moreover, as the content of MXene increases, the overall mechanical properties of the A@M_x_ film exhibit a trend of first increasing and then decreasing, with the A@M_3_ film showing optimal performance due to its highest MXene orientation degree of 0.89 (Figure 2f). This orientation enhances the effective transmission of stress, thereby improving mechanical properties. Notably, the mechanical properties in the X‐direction are slightly superior to those in the Y‐direction. At the same MXene content (33.7 wt.%), the X‐direction demonstrates higher tensile strength (121.0 ± 3.2 MPa), greater toughness (11.3 ± 1.6 MJ m^−^^3^), and increased fracture strain (13.4 ± 1.6%) compared to the Y‐direction (105.4 ± 7.7 MPa, 7.3 ± 0.9 MJ m^−^^3^, and 10.9 ± 0.4%, respectively). This anisotropy primarily stems from the directional growth of ice crystals during fabrication, leading to a well‐aligned ANF skeleton along the X‐direction, thereby enhancing the mechanical performance.

The reinforcement mechanism of the ANF skeleton in A@M_x_ films was examined through an analysis of both crack propagation and fracture surfaces. As illustrated in Figure 3d, the crack follows a tortuous propagation path, characterized by curled fracture edges, extracted MXene nanosheets, and bonded nanofibers. The “zigzag” fracture surfaces (Figure 3e; Figure S13) reveal that the damaged MXene layer remains concealed beneath this structure, while ANF layers experience stretching and slippage, resulting in prominent protrusions at the fracture surfaces. The tortuous crack path and “zigzag” fracture surface indicate that the A@M_x_ films can effectively absorb fracture energy, thereby enhancing mechanical performance [49]. Furthermore, to provide an intuitive insight into the fracture process, the stress distribution within the A@M_x_ films during stretching was methodically analyzed via finite element simulations (see Figure S1 for detailed simulation). As depicted in Figure 3f and Figure S14, during the initial stretching period, the applied force on the A@M_x_ films is primarily borne by the MXene layers due to their superior rigidity compared to the ANF layers, leading to remarkable stress concentration within the MXene layers, particularly at the film edges. However, as a result of the intrinsically weak mechanical properties (low strength and toughness) of these MXene layers, fractures initiate first within them at the stress concentration points when the applied strain exceeds their maximum bearing capacity. Subsequently, the ANF layers take over as the primary stress‐bearing component due to their higher strength and toughness. Ultimately, the A@M_x_ films undergo fracture failure starting from the film edges when the tensile strain surpasses the maximum allowable limit of the ANF layers.

### 2.3 EMI Shielding Performance of the Interpenetrating ANF/MXene Film

**FIGURE 4:**
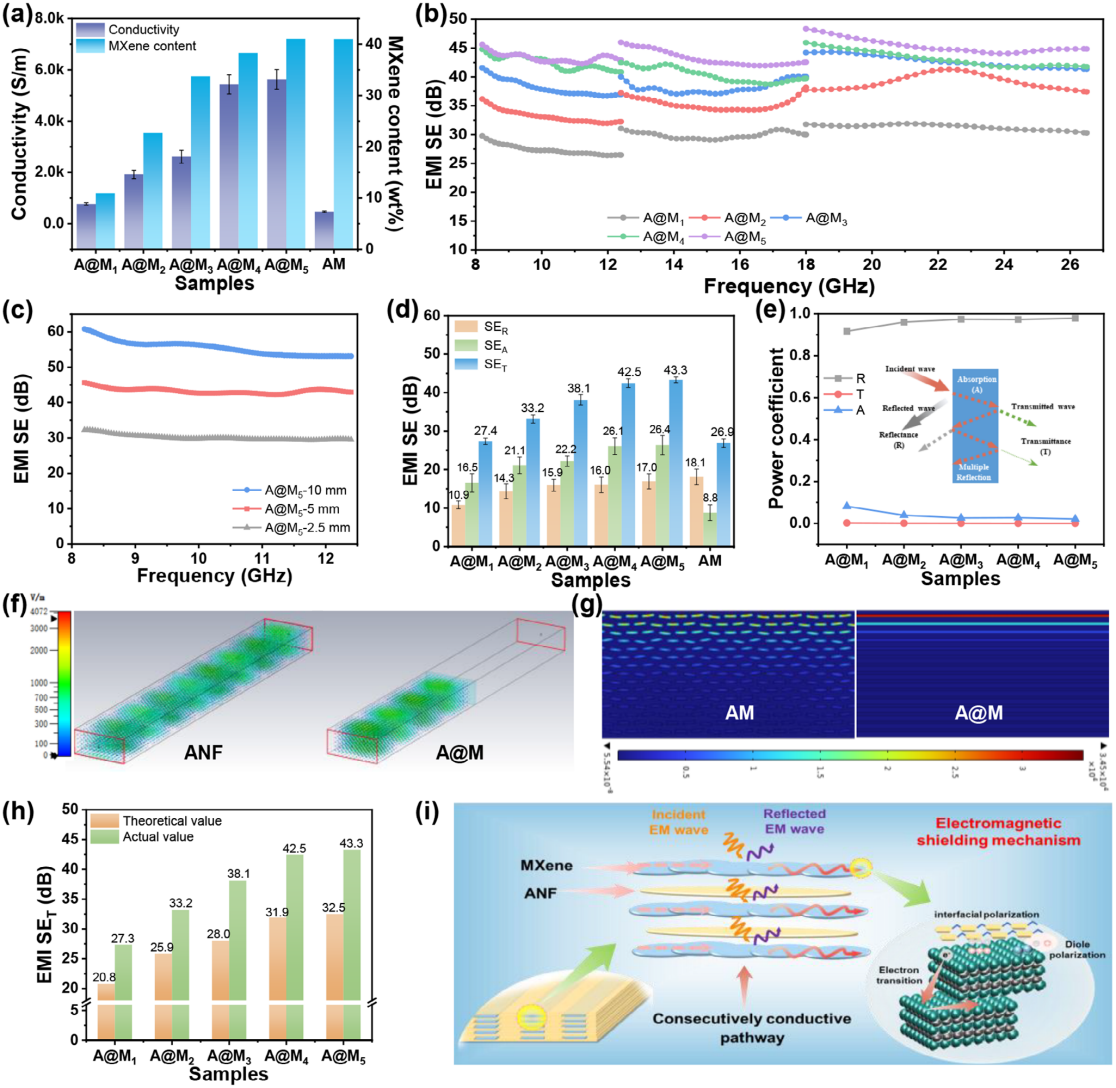
EMI shielding performance of interpenetrating ANF/MXene layered films: (a) Electrical conductivity and MXene content. EMI shielding efficiency curves (b) in X, Ku, and K bands, (c) with different gel thickness. (d) Average SE_T_, SE_R_, and SE_A_, and (e) average power coefficients of R, A, and T at X‐band. Finite element simulations for (f) electric field distribution when EMW traversing ANF and A@M films, and (g) polarization distribution in AM and A@M films. (h) Experimental and theoretical EMI shielding efficiency based on Schelkunnoffs’ formula. (i) Schematic illustration of EMI shielding mechanisms.

In the present work, the infiltration of MXene into the honeycomb pores incorporated into the formation of an interpenetrating and interconnected conductive network. This structure substantially reduced the contact resistance between conductive fillers, thereby greatly enhancing the electrical conductivity of the layered films. For instance, the interpenetrating A@M_5_ film demonstrates a remarkable conductivity of 5630.8 S/m, substantially surpassing that of the homogeneous AM film (472.4 S/m) with the same MXene content (Figure 4a). It should be noted that structural densification plays a crucial role in enhancing electrical conductivity and electromagnetic shielding performance. A densely compacted conductive layer provides sufficient contact between MXene nanosheets, thereby improving electron transport and EMI shielding effectiveness. Conversely, a loosely structured conductive layer leads to the opposite outcome (Figure S15).

The interpenetrating hierarchical structure fosters an abundance of interlaminar interfaces, which significantly enhances the multiple reflection and scattering of EMW, ultimately achieving exceptional EMI shielding performance even when the skin depth exceeds the film thickness (Figure S16). As anticipated, the A@M_x_ film displays broadband EMI shielding performance that progressively improves with increased MXene content and frequency (Figure 4b). Specifically, the average EMI shielding effectiveness (SE) rose from 27.4 to 43.3 dB as MXene content increased from 10.9 to 41.1 wt.% and further advanced from 43.3 dB (X‐band) to 45.4 dB (K‐band) for the A@M_5_ film. Additionally, by augmenting the film thickness, the EMI SE of A@M_5_ film can be further enhanced to 55.5 dB (Figure 4c). When comparing the EMI shielding performances of A@M_x_ and AM films in X‐band at the same MXene content (Figure 4d), the A@M_5_ film demonstrates significantly higher average SE_R_, SE_A_, and SE_T_ values of 17.0, 26.4, and 43.3 dB, respectively, while the AM film only reaches 18.1, 8.8, and 26.9 dB. The substantial enhancement in SE_A_ highlights the contribution of the interpenetrating and interconnected MXene network in amplifying SE_T_ through multiple reflection and scattering absorption mechanisms. Notably, while MXene layers may form a parallel array, the A@M film exhibits nearly consistent EMI shielding performance in both directions parallel and vertical to the ice crystal growth (Figure S17). This uniformity arises because the internal pores of the unidirectional ANF honeycomb hydrogel are not fully enclosed but are instead interconnected. Consequently, the MXene incorporated during immersion creates an interconnected conductive network, resulting in isotropic EMI shielding.

Furthermore, the SE_R_, SE_A_, R, and A power coefficients were computed using Equations (5)–(8) to elucidate the EMI shielding mechanism. The results reveal that SE_R_ and SE_A_ gradually rise from 10.9 and 16.5 dB to 17.0 and 26.4 dB, respectively, as the MXene content increases (Figure 4d). These findings indicate that higher electrical conductivity enhances both reflection loss due to increased impedance mismatch and absorption loss through ohmic dissipation and repeated internal reflections. Notably, the SE_R_ values ranging from 10–20 dB for all samples imply that over 90%–99% of the incident radiation is obstructed before entering the samples. In conjunction with the R‐A power coefficients analysis (Figure 4e), the significantly higher R values (0.917–0.979) compared to the corresponding A values (0.081–0.021) suggest that the reflection shielding mechanism predominates in the A@M_x_ films. In addition, the theoretical SE values derived from Schelkunoff's equations are consistently around 10 dB lower than the experimental measurements for all A@M films (Figure 4h). As noted by Zhang et al., this observed discrepancy primarily originates from insulating structures that extend beyond the conductive network [1]. In reality, the interpenetrating MXene layers within the ANF honeycomb architecture contribute to the formation of numerous micro‐capacitors in the A@M_x_ films, which generate alternating micro‐currents and opposing induced EM fields in response to EMW exposure, thereby contributing to EMI shielding mechanisms beyond conventional conductive effects.

CST Microwave Studio was utilized to quantitatively assess the shielding effects of the pure ANF, AM, and A@M_5_ films at the macroscopic level, as shown in Figure 4f and Figures S18–S20 (see Figure S2 for a detailed simulation). The comparison indicates that pure ANF films exhibit minimal electromagnetic shielding effectiveness, allowing a majority of EMW to pass through unimpeded. Conversely, the MXene‐based layered films (AM, A@M) demonstrate exceptional microwave attenuation capabilities, effectively blocking nearly all incident EMW on one side of the films. Notably, the A@M_5_ film exhibits a stronger shielding effect compared to the AM film, attributable to its higher EMW reflection and absorption resulting from elevated conductivity and the interpenetrating layered structure. To further explore the influence of MXene's spatial distribution within the layered films on EMW loss capacity, finite element analysis was conducted utilizing COMSOL software to simulate the electric‐magnetic field distributions, polarization, and power loss at the microscale level in both AM and A@M_5_ films (see Figure S2 for detailed simulation). As illustrated in Figure 4g and Figures S21 and S22, when EMWs strike the AM film, they rapidly induce strong surface polarization and weak magnetization in the MXene nanosheets, resulting in substantial EMW power loss. Both polarization and power loss gradually diminish as the waves propagate deeper into the AM film. Consequently, both electric and magnetic field intensities exhibit a monotonically decreasing trend from the surface toward the interior of the AM film. In contrast, for A@M films, the polarization and magnetization induced by alternating electromagnetic waves primarily occur within the initial few MXene layers (particularly the first layer) that first interact with the waves, leading to the corresponding power loss localized in these MXene layers. Thus, due to the exceptional EMW loss capability of the surface interpenetrating MXene layers, the electromagnetic field within the A@M film exhibits a cliff‐like drop in distribution beyond the surface MXene layers. Therefore, the simulations reveal that the interpenetrating MXene network exhibits superior polarization and EMW loss capabilities compared to uniformly distributed MXene nanosheets, thereby facilitating more effective EMI shielding.

Consequently, based on the preceding analyses, we propose a potential EMI shielding mechanism grounded in the transmission line model, as illustrated in Figure 4i. Initially, the impedance mismatch between the A@M_x_ conductive film and free space results in substantial reflection of EMWs at the interface [50, 51]. Subsequently, the EMWs that infiltrate the interior of the films engage with the high‐density electron network of MXene via multiple mechanisms, including interface polarization, dipole polarization, conductive loss, and eddy current effects, among others [52]. These interactions also generate opposing induced EM fields in the pseudo–parallel plate micro‐capacitors formed by the interpenetrating MXene and ANF layers. Collectively, these mechanisms convert EM energy into thermal energy, facilitating efficient absorption loss [53]. Crucially, the unique interpenetrating network intensifies the multiple reflection and scattering of EMWs between the MXene and ANF layers [54], thereby amplifying the aforementioned absorption loss mechanisms, resulting in virtually no EMW transmission through the A@M_x_ films. Therefore, our A@M_x_ films exhibit comprehensive EMI shielding performance in comparison to reported shielding films, characterized by their low MXene loading, superior mechanical properties, and optimal thickness (Table S2).

### 2.4 Antioxidation and Durability of the Interpenetrating ANF/MXene Film

**FIGURE 5:**
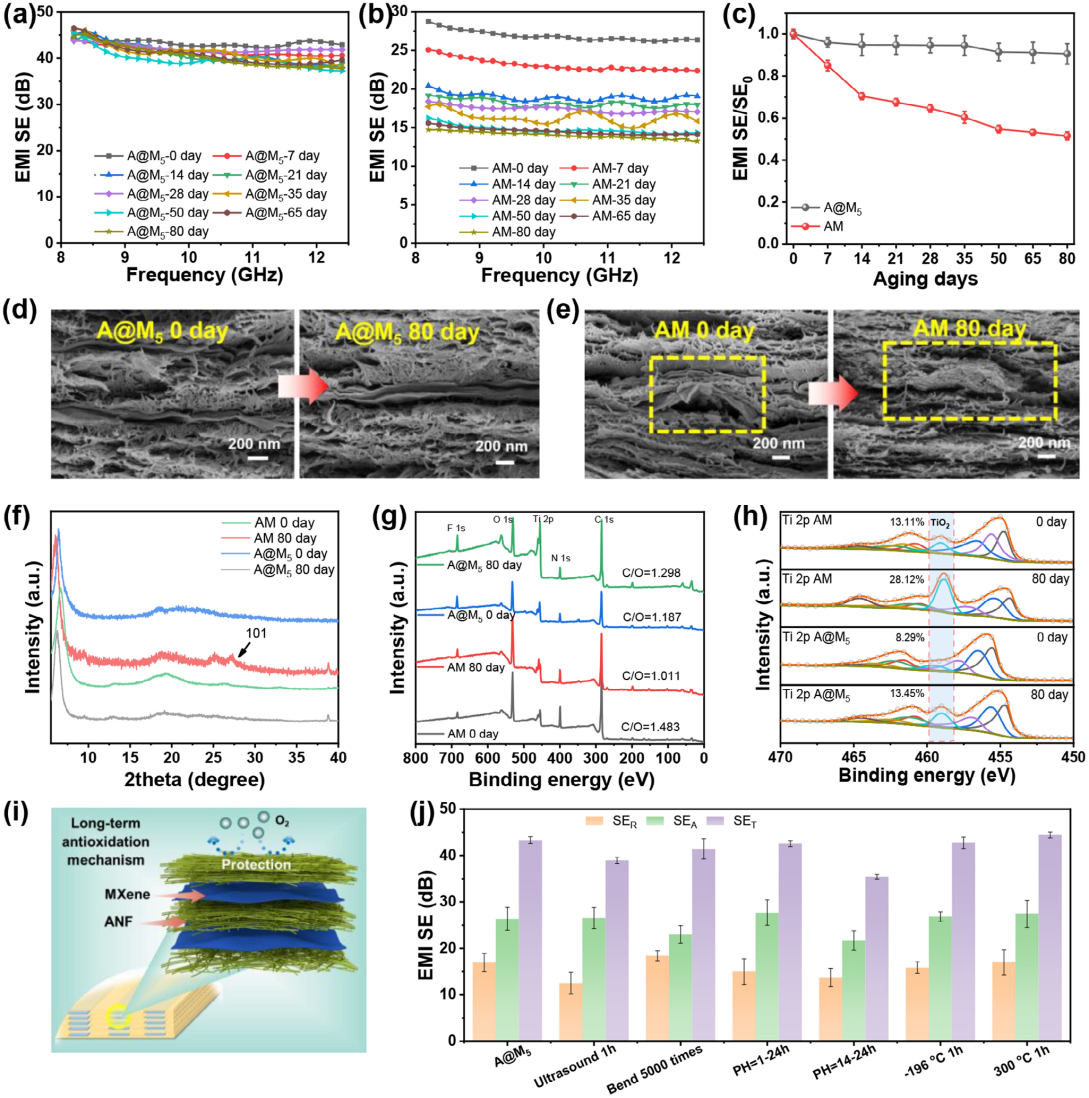
Long‐term antioxidation property of the interpenetrating ANF/MXene layered films: EMI shielding efficiency curves in X band of (a) A@M_5_ and (b) AM films with different aging days, and (c) the corresponding average SE_T_ as a function of aging days. Cross‐sectional SEM images of (d) A@M_5_ and (e) AM films before and after aging treatment. (f) XRD patterns, (g) XPS survey, and (h) high‐resolution XPS spectra at Ti 2p region of A@M_5_ and AM films before and after aging treatment. (i) Schematic illustration antioxidation mechanism of A@M_x_ films. (j) Comparison of the average SE of A@M_5_ film before and after different durability treatments.

Generally, the intrinsic oxidative instability of MXene significantly undermines the antioxidant capacity of its composites, which poses a critical barrier to their practical applications [55, 56, 57]. In an interpenetrating layered structure, the honeycomb‐formed ANF layers shield the conductive layers from air exposure, thereby markedly enhancing oxidation resistance. As illustrated in Figure 5a–c, our interpenetrating A@M_5_ film exhibits almost unchanged EMI shielding performance even after 80 days of air exposure, despite an increase in sheet resistance from 5.1 to 34.0 Ω/sq due to surface MXene oxidation (Figure S23a). In stark contrast, the homogeneous AM film, produced via vacuum‐assisted filtration, demonstrates an approximate 50% reduction in EMI SE, coupled with a dramatic rise in sheet resistance from 68.8 to 1334.3 Ω/sq (Figure S23b). This striking disparity highlights how the interpenetrating layered structure significantly bolsters the antioxidant stability of the MXene‐based film.

To methodically examine the mechanisms that underpin antioxidant stability, we conducted a comparative analysis of morphological and physicochemical structural alterations in the films prior to and following extended air exposure. As demonstrated in Figure 5d,e, the A@M_5_ film retains a smooth and intact morphology with well‐preserved interpenetrating MXene nanosheets even after 80 days of air exposure, exhibiting no detectable formation of oxidized particles. In sharp contrast, the AM film displays significant surface oxidation after the same duration, with numerous particles conspicuously visible on the MXene nanosheets—a dramatic deviation from its initial smooth surface morphology. The particles observed on the MXene surfaces are hypothesized to be TiO_2_ particles resulting from oxidation. To validate this hypothesis, XRD and XPS analyses were conducted on the films before and after air exposure. According to Figure 5f, the XRD spectrum of the AM film after air exposure reveals a characteristic peak associated with the (101) plane of anatase TiO_2_, whereas the A@M_5_ film maintains nearly identical XRD patterns before and after exposure. The XPS survey indicates that the C/O ratio of the AM film significantly decreases from 1.483 to 1.011 after exposure (indicative of a substantial increase in O content due to oxidation), while the C/O ratio of the A@M_5_ film illustrates only a slight increase (Figure 5g). From the XPS spectra in the Ti 2p region, it is evident that the TiO_2_ peak area ratio in the AM film rises considerably from 13.11% to 28.12% after 80 days of air exposure, whereas that in the A@M_5_ film displays only a modest increase from 8.29% to 13.45% (Figure 5h).

Therefore, based on the analyses presented, it can be concluded that the limited antioxidant capacity of the AM film primarily arises from the homogeneous dispersion of MXene nanosheets, which are susceptible to oxidation into TiO_2_ upon exposure to oxygen. This oxidation drastically reduces the intrinsic electrical conductivity of MXene, leading to a notable decline in the film's EMI shielding performance. In contrast, the A@M_x_ films feature interpenetrating MXene layers that are entirely encapsulated within an ANF matrix formed through honeycomb wall densification. This complete encapsulation effectively protects the MXene conductive network from atmospheric exposure, thereby preventing oxidation during practical applications. As a result, the A@M_x_ films demonstrate exceptional antioxidant EMI shielding performance (Figure 5i). Furthermore, the interpenetrating layered structure enables the A@M film to maintain stable EMI shielding performance under various extreme conditions, including ultrasonic treatment, bending, acidic or alkaline environments, extreme temperatures, hygrothermal conditions, and UV radiation (Figure 5j; Figures S24 and S25). Besides, the A@M film endures 1000 bending cycles without significant degradation in EMI SE, showcasing its excellent durability and mechanical flexibility (Figure S26). Therefore, the excellent antioxidant properties and stability ensure the durability of A@M_x_ film in practical applications.

## 3 Conclusion

In summary, we developed an innovative directional freezing–thaw intercalation–gel‐film formation technique to fabricate A@M films with an interpenetrating layered structure. The ice‐templated pores facilitated the organized and interconnected arrangement of MXene sheets, establishing coherent conductive pathways. Simultaneously, a unique “filler‐matrix‐filler” micro‐capacitor structure was constructed, significantly enhancing EMI shielding performance. Notably, the A@M film achieved an extraordinary EMI SE of 43.3 dB, vastly exceeding the 26.9 dB of the vacuum‐assisted filtration film. The interpenetrating layered structure, in which the ANF matrix encapsulates the MXene layers, could effectively obstruct H_2_O and O_2_ from reacting with MXene, thereby ensuring long‐term oxidation stability. Even after 80 days of air exposure, the EMI shielding performance experienced only a minimal degradation of 10%. Additionally, the interpenetrating ANF layers formed a robust mechanical framework, granting the A@M film exceptional mechanical properties. With its flexibility, mechanical robustness, broad‐spectrum EMI shielding capability, and oxidation resistance, this interpenetrating layered film holds substantial promise for practical applications in advanced EMI shielding.

## 4 Experimental Section

### 4.1 Preparation Process of ANF/MXene Layered Film with Interpenetrating Conductive Networks (A@M film)

First, aramid nanofiber (ANF, 5 mg/mL) was prepared by a ball‐milling assisted deprotonation process for Kevlar fiber (K49, Dupont) in a DMSO/H_2_O/KOH system reported by our previous work [58]. The successful preparation of ANF was verified by optical microscopy, FTIR spectroscopy, and morphological characterization via TEM and SEM imaging (Figure S27). Ti_3_C_2_T_x_ MXene nanosheets (1–5 mg/mL in aqueous solution) were obtained by etching Ti3AlC2 powders (MAX, 200 mesh, 11 Technology) by the LiF/HCl system, and the detailed process was seen in our previous work [59]. The successful preparation of MXene was verified by the structure analysis vis XRD and XPS spectroscopy, and morphological characterization via TEM and SEM imaging (Figure S28).

Then, the ANF solution was poured into a rectangular plastic mold with a copper bottom immersed in liquid nitrogen for directional freezing casting. After completely frozen, the frozen ANF block with directional ice structure was put into anhydrous ethanol (−20°C) for solvent replacement. This process was repeated three times until DMSO is completely replaced by ethanol, thereby obtaining an ANF hydrogel with directional pore structure. Subsequently, the ANF hydrogel was immersed in MXene aqueous solution (80 mL, 1–5 mg/mL) and soaked under vacuum conditions (vacuum oven, room temperature) for 24 h. After that, the obtained ANF/MXene hydrogel was squeezed by a 5 kg weight to remove most of the water, then was hot‐pressed and dried under 10 MPa and 100°C for 4 h by using a press machine, finally obtaining the ANF/MXene layered film with interpenetrating conductive networks. For simplicity, the resulting films are named A@M*x*, where *x* stands for ANF gel immersed in *x* mg/mL MXene solution. The comparison sample, ANF/MXene layered film (AM) with homogeneous layer structure, was obtained by direct vacuum‐assisted filtration of ANF/MXene mixed solution.

### 4.2 Characterizations

Microscopic morphology and structure were observed by SEM (Zeiss Sigma 300) with an accelerating voltage of 20 kV, equipped with an energy‐dispersive spectrometer (EDS). XRD patterns were obtained by a Rigaku Ultima IV diffractometer coupled with Cu Kα radiation (λ = 0.154 nm). WAXD analysis was conducted on a Bruker D8 Discovery X‐ray setup equipped with a Cu‐Kα source. FTIR spectra were recorded by a Nicolet NEXUS 6700 spectrometer based on the angle of total reflection model. XPS analysis was carried out on a Thermo‐ESCALAB 250X spectrometer. TGA analysis was executed on a NETZSCH STA 209F3 device in a nitrogen atmosphere with a heating rate of 10°C min^−1^. A universal stretching testing machine (SUNS UTM2203) was used to measure the mechanical properties. The electrical conductivity was tested by a four‐probe resistivity measurement system (RTS‐8). The EMI shielding performance in X, Ku, and K bands was evaluated on a vector network analyzer (Agilent N5244A). The scattering parameters of S_11_ and S_22_ were measured by the waveguide method with the sample sizes of 22.86 × 10.16 mm^2^ for X‐band, 15.80 × 7.90 mm^2^ for Ku‐band, and 10.70 × 4.30 mm^2^ for K‐band, and the power coefficients (A, R, T) and EMI shielding effectiveness (SE) are calculated according to the following equations:

(5) $$R={\left|S_{11}\right|}^{2},\;T={\left|S_{21}\right|}^{2}$$

(6) A=1−R−T

(7) $$SE_{R}=-10\log\;\left(1-R\right),\;SE_{A}=-10\log\left(\frac{T}{1-R}\right)$$

(8) $$SE_{T}=SE_{R}+SE_{A}+SE_{M}$$

where A, R, and T are the coefficients of reflectivity, absorptivity, and transmission, respectively, and SE_T_, SE_R_, SE_A_, and SE_M_ are the total shielding effectiveness, microwave reflection, microwave absorption, and multiple reflection, respectively. SE_M_ can usually be ignored when SE_T_ > 15 dB.

## Conflicts of Interest

The authors declare no conflicts of interest.

## Supporting information




**Supporting File**: advs73949‐sup‐0001‐SuppMat.docx.

## Data Availability

The data that support the findings of this study are available in the supplementary material of this article.
