# Supplementary material for: Engineered Interpenetrating MXene Networks in Aramid Layered Films for Antioxidant and Broadband Electromagnetic Interference Shielding
Source: Adv Sci (Weinh). 2026 Jan 20;13(18):e20793. doi: 10.1002/advs.202520793 (PMC13042854; doi:10.1002/advs.202520793)
Supplement: Supplementary file 1 — Supporting File: advs73949‐sup‐0001‐SuppMat.docx. [file ADVS-13-e20793-s001.docx]

Supporting Information

**Engineered Interpenetrating MXene Networks in Aramid Layered Films for Antioxidant and Broadband Electromagnetic Interference Shielding**

*Hongli Cheng*^1,†^*, Gaojie Han*^1,†^*, Congqi Liu*^1^*, Yang Zhou*^1^*, Yiming Wang*^1^*, Bing Zhou*^1^*, Yuezhan Feng*^1^,*^*^, Chuntai Liu*^1^*, Hao-Bin Zhang*^2^,*^*^, Changyu Shen*^1^

^1^State Key Laboratory of Structural Analysis Optimization and CAE Software for Industrial Equipment, National Engineering Research Center for Advanced Polymer Processing Technology, Zhengzhou University, Zhengzhou 450002, China.

^2^State Key Laboratory of Organic-Inorganic Composites, College of Materials Science and Engineering, Beijing University of Chemical Technology, Beijing 100029, China.

^†^ Hongli Cheng and Gaojie Han contributed equally to this work.

^*^ Correspondence: Yuezhan Feng ([yzfeng@zzu.edu.cn](mailto:yzfeng@zzu.edu.cn)), Hao-Bin Zhang ([zhanghaobin@buct.edu.cn](mailto:zhanghaobin@buct.edu.cn))

**S1. Simulation of Mechanical Properties**

The tensile fracture simulation of A@M_x_ films is carried out. Based on the objective situation that the film thickness is uniform, the 2D simplified geometric models of the three structures are created respectively, surface stress assumption is used in the simulation. The geometric model was constructed by rectangle, array and bourg calculation tools. The total thickness of the film was uniform, which was 30 μm, and the volume fraction of MXene in the film structure was 40%. The length and width of the stretched film were 15 mm × 3 mm. The materials are mainly ANF and MXene, in which the input Young's modulus of the ANF is 2.64343 GPa, the Poisson's ratio is 0.35, the density is 1440 kg/m^2^, and the critical fracture stress is 132.05595 MPa. MXene material properties, input Young's modulus 0.82958 GPa, Poisson's ratio 0.3, density 4200 kg/m^2^, critical fracture stress 9.84717 MPa. The physical field is solid mechanics, the linear elastic material and damage condition are added, and the phase field damage model is used. The principal stress criterion is used as the crack driving condition, and the other parameters are from the material. The steps are as follows:

The film thickness is much smaller than other dimensions, so the plane stress condition is satisfied:

$\sigma_{33}=0$ (S1)

So the stress-strain relationship is:

$\sigma_{11}=\frac{E}{1-\upsilon^{2}}\left(\varepsilon_{11}+\upsilon\varepsilon_{22}\right)$ (S2)

$\sigma_{22}=\frac{E}{1-\upsilon^{2}}\left(\varepsilon_{22}+\upsilon\varepsilon_{11}\right)$ (S3)

$\sigma_{12}=2G\varepsilon_{12}$ (S4)

Where *E* is the elastic modulus and *υ* is the Poisson ratio. Where *G* is the shear modulus and $G=\frac{E}{2\left(1+\upsilon\right)}$.

According to the tensile test of the material, the kinematic relationship is consistent with the small deformation theory:

$\varepsilon_{ij}=\frac{1}{2}\left(\frac{\partial u_{i}}{\partial x_{j}}+\frac{\partial u_{j}}{\partial x_{i}}\right)$ (S5)

Where *ε_ij_* is the strain tensor, *u_i_* is the displacement component, and *x_i_* is the initial coordinate.

The material model uses a linear elastic material and obeys Hooke's law:

$\sigma_{ij}=C_{ijkl}\varepsilon_{kl}$ (S6)

Where σ*_ij_* is the stress tensor and *C_ijkl_* is the elastic stiffness matrix.

Because of the steady-state solution, the equilibrium equation is momentum conservation:

$\nabla\cdot\sigma+f=0$ (S7)

Where *f* is the external force.

The damage failure criterion uses the maximum principal stress criterion:

$\sigma_{1}\geq\sigma_{crit}$ (S8)

The σ_1_ is principal stress. Here, due to the planar stress assumption$\sigma_{1}=\frac{\sigma_{11}+\sigma_{22}}{2}+\sqrt{\left(\frac{\sigma_{11}+\sigma_{22}}{2}\right)^{2}+\sigma_{12}^{2}}$. σ_crit_ is the tensile strength of the material.

Principle of virtual work:

$\int_{\Omega} \sigma\text{:}\delta\varepsilon d\Omega=\int_{\partial\Omega} t\cdot\delta ud\Gamma$ (S9)

Taking into account the relationship between strain and displacement and the constitutive model of the material, and substituting the principle of virtual work, the element stiffness matrix of the basic equation for finite element analysis can be obtained:

$K^{e}=\int_{\Omega^{e}} B^{T}Dd\Omega$ (S10)

B is the strain-displacement matrix and D is the matrix derived from the constitutive equation (*σ=Dε*).

The film is discretized into finite elements, and the strain of the element is calculated by the displacement of the nodes, and the stress is calculated according to equation S6. The damaged elements are marked according to the damage failure criterion formula S8. Finally, the stiffness matrix of the overall assembly element is a global matrix. Combined with the external force field generated by the boundary conditions, a new displacement field can be obtained by solving the equilibrium equation S7 and so on.

The initial value of the displacement field is set to 0, and the specified displacement conditions are set at both ends of the stretching spline. The left end specifies that the x-direction displacement is 0 and the Y-axis direction is free. The right-hand side specifies the x-axis displacement as Delta, and sets Delta to 0 [mm] in the parameter setting, with the steady-state study setting step adding an auxiliary scanning condition range (0, 0.01, 0.5). To achieve the condition of stretching to the right. That is, set the boundary conditions to the left *u_x_*=0, the right *u_x_*=*Delta*, and the Delta changes gradually according to the auxiliary scan conditions.

**S2. Electromagnetic Simulation Method**

The macroscopic electromagnetic analysis was performed in CST STUDIO SUITE 2020. The geometry was modeled as a 22.86 mm × 10.16 mm × 140 mm rectangular block. The computational domain was bounded by 0 mm in ±X and ±Y, and by 80 mm in ±Z. Boundary conditions assigned electric walls (Et = 0) to the four lateral faces, while the top and bottom surfaces received open (add space) treatment. A broadband sweep from 8.2–12.4 GHz was executed with a tetrahedral mesh. Two waveguide ports, oriented antiparallel along Z, fully covered the respective cross-sections, with excitation applied to Port 1 in multimode operation. Monitors captured E-field, H-field, power, and surface currents at 10.3 GHz. The film model was centrally positioned within the waveguide cavity. The thicknesses of composite films A@M_5_, AM, and pure ANF are all 2 mm, respectively. Material parameters for all components were defined using either the raw datasets obtained from different samples.

The electromagnetic loss performance of MXene at the microscopic level was analyzed using COMSOL Multiphysics software. The radio frequency mode was selected use finite element simulation programs. In the perfectly matched layer an electromagnetic wave with a frequency of 10 GHz was imported and corresponding conductivities of MXene and ANF are set to be the same in Figure 4d. The wave transmission is shown as follows:

$\nabla\times\mu_{r}^{-1}\left(\nabla\times E\right)-k_{0}^{2}\left(\varepsilon_{r}-\frac{j\sigma}{\omega\varepsilon_{0}}\right)E=0$ (S11)

$E\left(x,y,z\right)=\widetilde{E}\left(x,y\right)e^{-ik_{Z}Z}$ (S12)

Floquet periodic boundary conditions:

$E_{dst}=E_{src}e^{-ik_{F}\cdot\left(r_{dst}-r_{src}\right)}$ (S13)

$H_{dst}=H_{src}e^{-ik_{F}\cdot\left(r_{dst}-r_{src}\right)}$ (S14)

The port of the electric field:

$S=\frac{\int_{\partial\Omega} \left(E-E_{1}\right)\cdot E_{1}}{\int_{\partial\Omega} E_{1}\cdot E_{1}}$ (S15)

The port of the magnetic field:

$S=\frac{\int_{\partial\Omega} \left(H-H_{1}\right)\cdot H_{1}}{\int_{\partial\Omega} H_{1}\cdot H_{1}}$ (S16)

the scattering boundary condition:

$n\times\left(\nabla\times\left(E\right)\right)\text{-}jkn\times\left(E\times n\right)\text{=}0$ (S17)

Ideal electrical conductor:

$n\times E\text{=}0$ (S18)

The typical wavelength definition of a perfectly matched layer in a fictional domain is:

$2*pi/abs(emw.k_{0}*cos(theta))$ (S19)

Among, **E**, **H**, *k*_0_, $\boldsymbol{\varepsilon}_{r}$, $\sigma$, $\omega$, $\varepsilon_{0}$, and theta represent the electric field intensity (V m^-1^), the magnetic field intensity (A m^-1^), the free space wave number (rad m^-1^), the complex dielectric constant (F m^-1^), the conductivity (S m^-1^), the vacuum dielectric constant (8.8542×10^-12^ F m^-1^) and incidence angle (rad), respectively.

The EMI shielding material model was established which contains the port layer, the air layer, and composite films, respectively. The upper edge of the air layer is a perfectly matched layer (PML) and an electric or magnetic field source port, the bottom edge is the scattering boundary layer (SBL) condition, and both sides are Floquet periodic boundary conditions. The EMW excitation source is the electric field polarization source or the magnetic field magnetization source along the Z-direction with an incident power set to 1 W m^-1^. The electric field and magnetic field at excitation source are vector orthogonal, and their phase difference is π/2.

**S3. Figures**


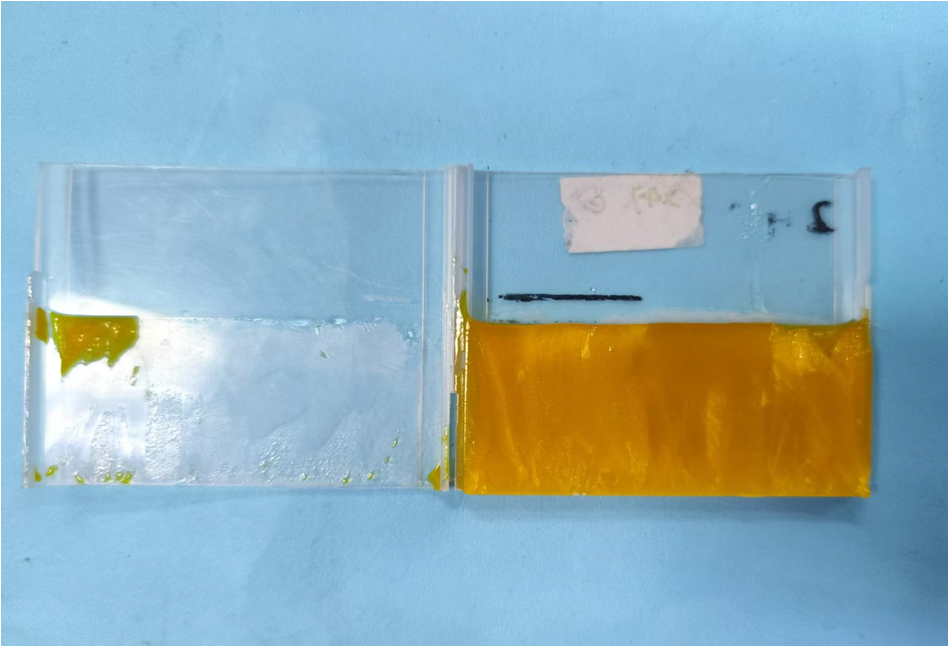


**Figure S1**. Photograph of the unidirectional frozen ANF block.


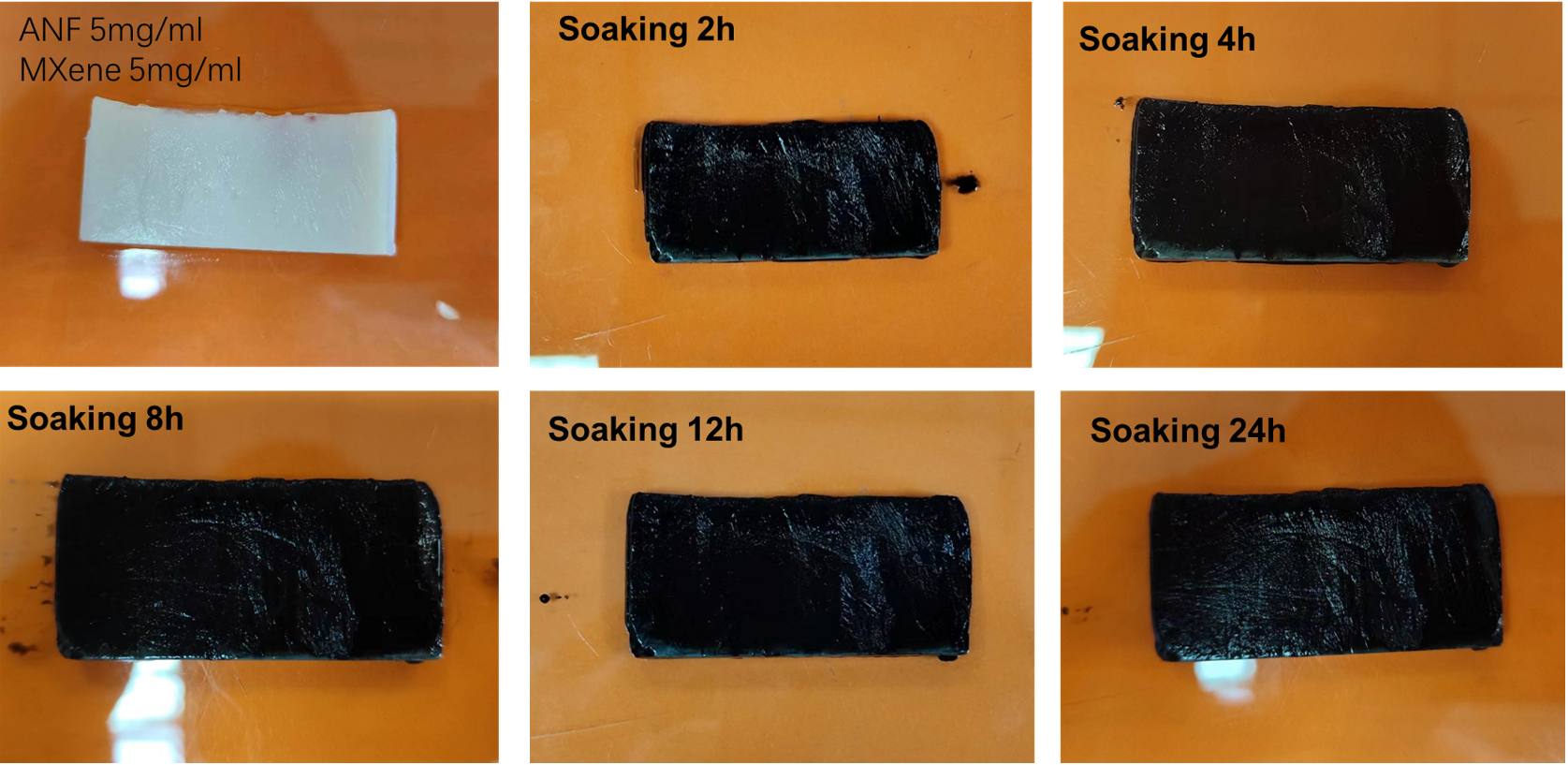


**Figure S2.** Photographs of the unidirectional ANF gels soaking in MXene solution with different time.


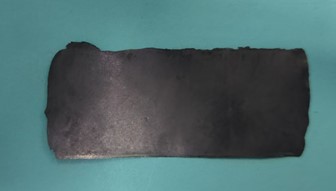


**Figure S3.** Photograph of A@M film after pressure-assisted densification.


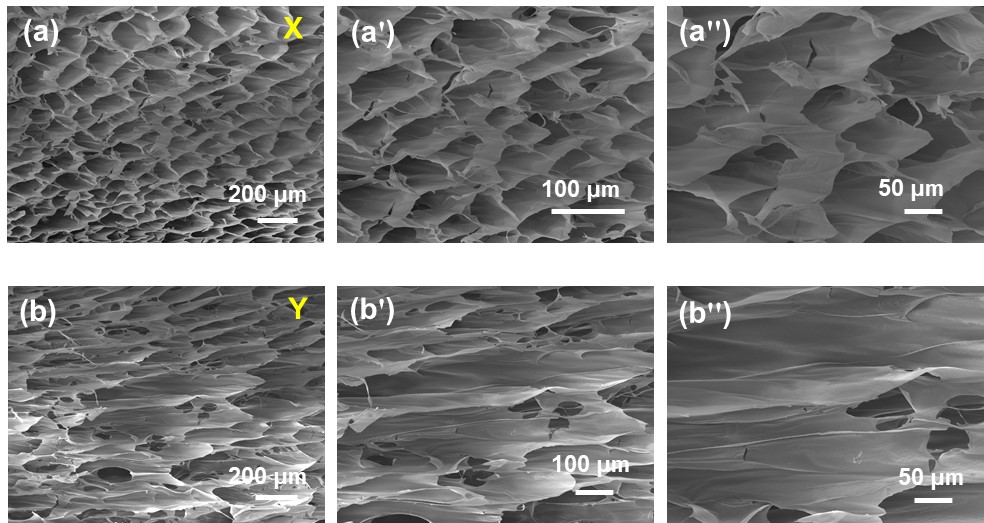


**Figure S4.** Cross-sectional SEM images in (a) X and (b) Y directions for the unidirectional ANF gel with different magnification.


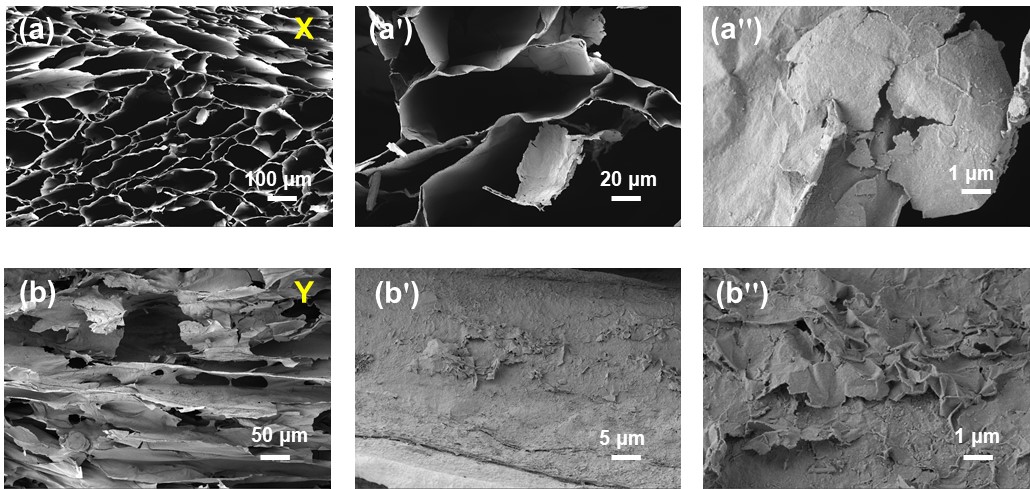


**Figure S5.** Cross-sectional SEM images in (a) X and (b) Y directions for the unidirectional ANF/MXene gel with different magnification.


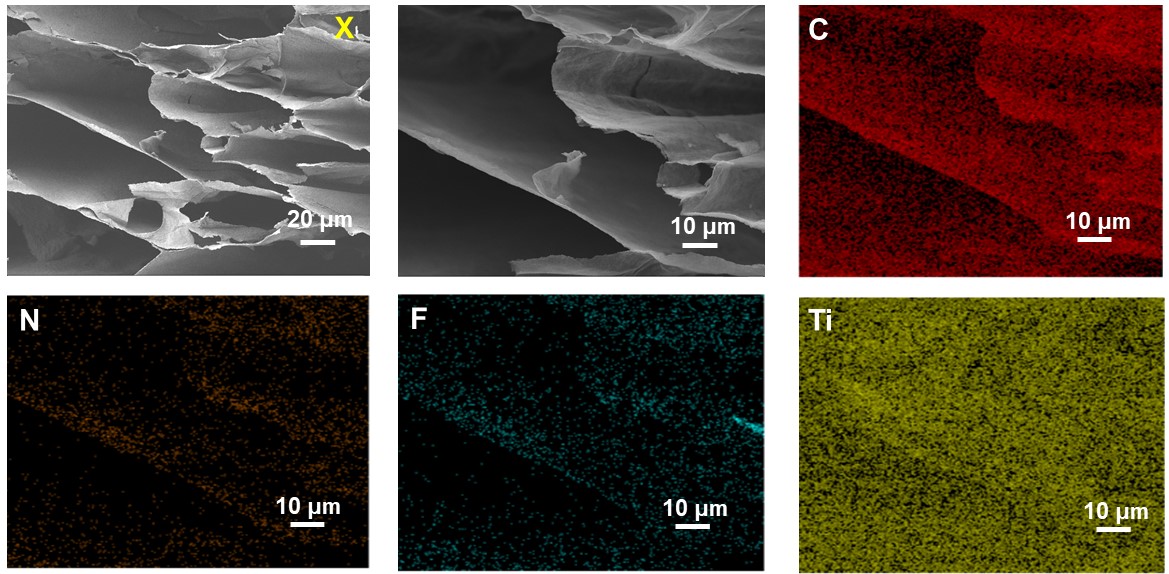


**Figure S6.** Cross-sectional SEM images and corresponding EDS mapping images for the unidirectional ANF/MXene gel.


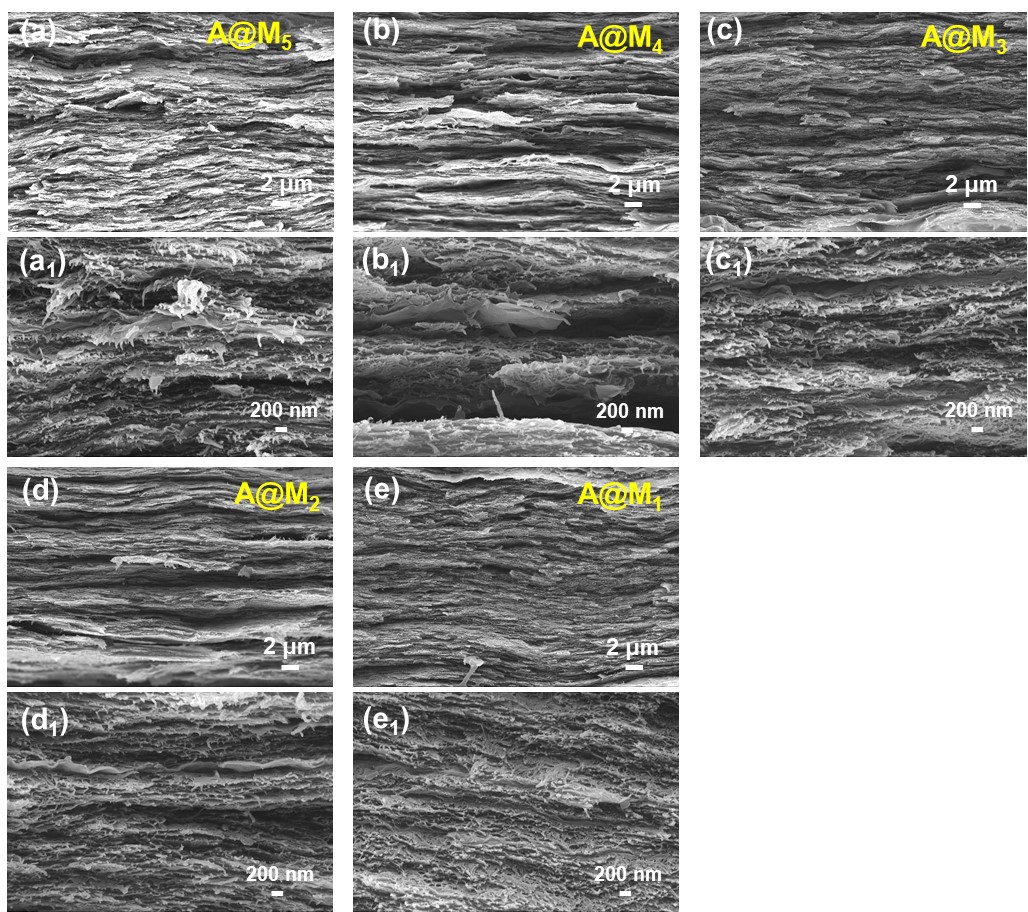


**Figure S7.** Cross-sectional SEM images for A@M_x_ films with different MXene content.


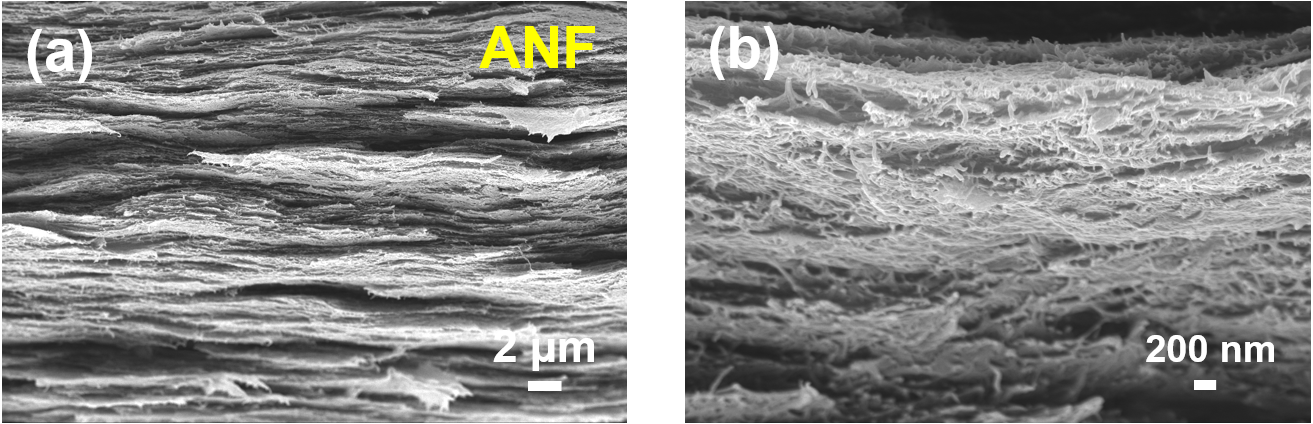


**Figure S8.** Cross-sectional SEM images of pure ANF film.


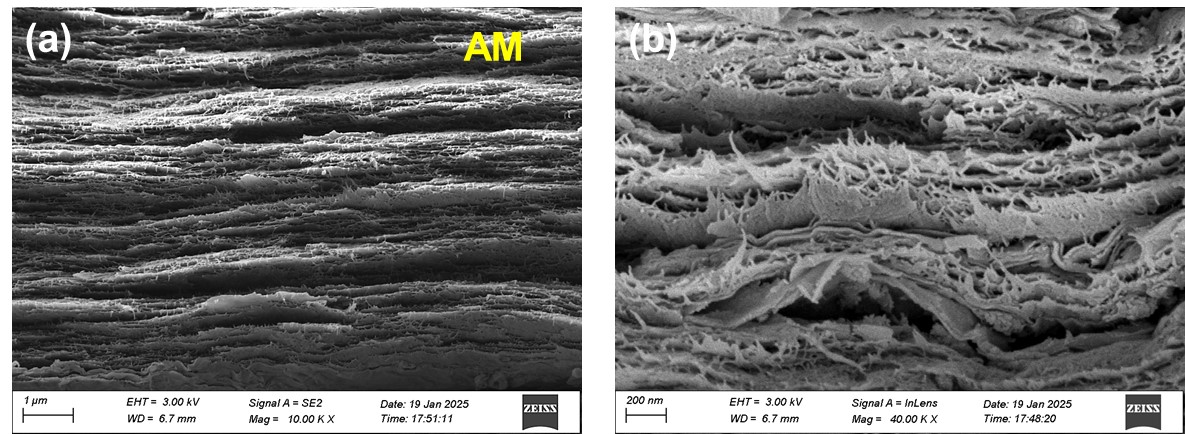


**Figure S9.** Cross-sectional SEM images of AM film.

**Figure S10.** DTG curves of ANF, MXene and A@M films with different MXene content.

**Figure S11.** FTIR spectra of ANF, MXene and A@M films with different MXene content.

**Figure S12.** (a) Photograph of A@M film for X and Y directions. (b) Stress-strain curves, (c) tensile strength and modulus, (d) toughness and fracture strain of ANF and A@M films along Y direction.


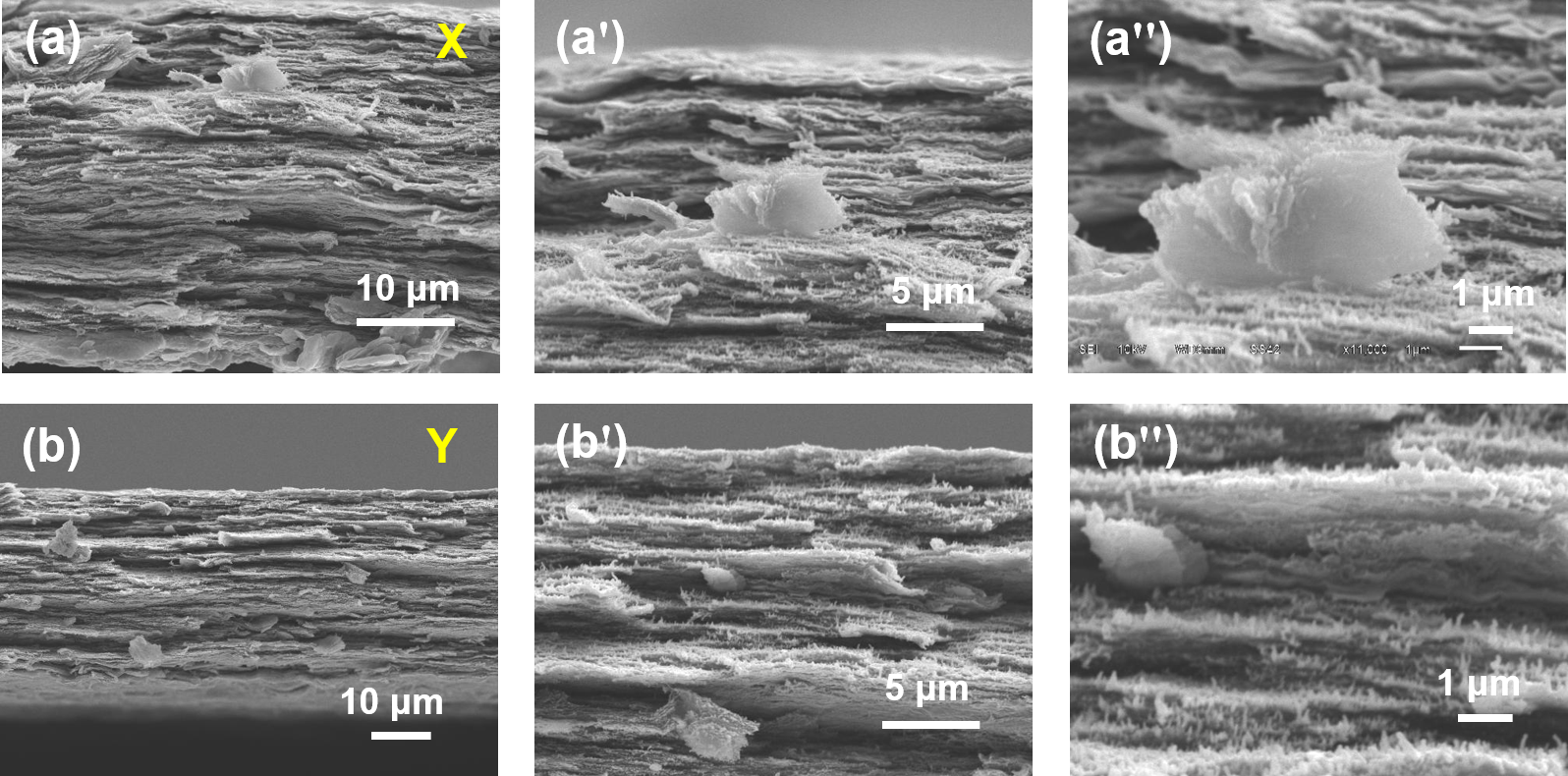


**Figure S13.** SEM images for the tensile fracture surface of A@M film along (a) X and (b) Y direction.

**Figure S14.** Finite element simulations of stress distribution for A@M_x_ film at different stretching stages.

**Figure S15.** (a-c) SEM images and (d) thickness distribution of the interpenetrating ANF/MXene films at different density thickness. (e) Sheet resistance and electrical conductivity of ANF/MXene films as a function of thickness, and (f) their EMI shielding effectiveness.

**Figure S16.** Skin depth of A@M films.


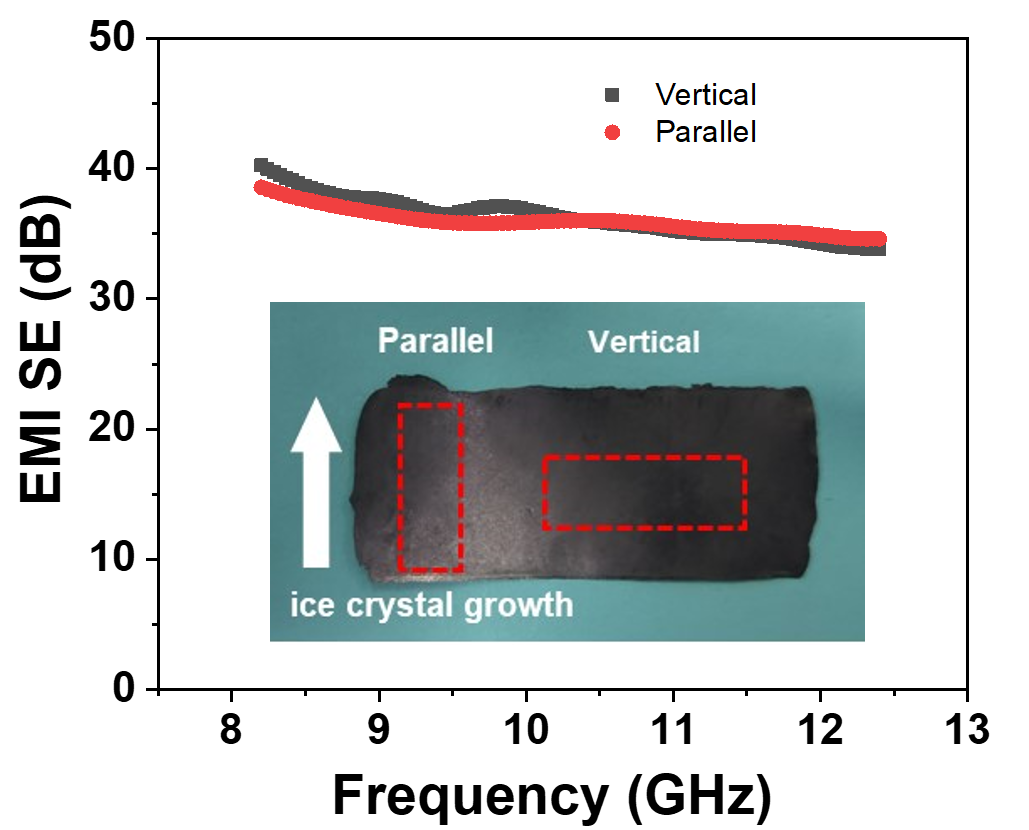


**Figure S17**. EMI shielding performance of A@M_4_ film along vertical and parallel directions of ice crystal growth.

**Figure S18.** Finite element simulations for magnetic field distribution when EMW traversing ANF and A@M films

**Figure S19.** Finite element simulations for power flow density distributions when EMW traversing ANF and A@M films.

**Figure S20.** Finite element simulations for electric field, magnetic field and power flow density distributions when EMW traversing AM film.


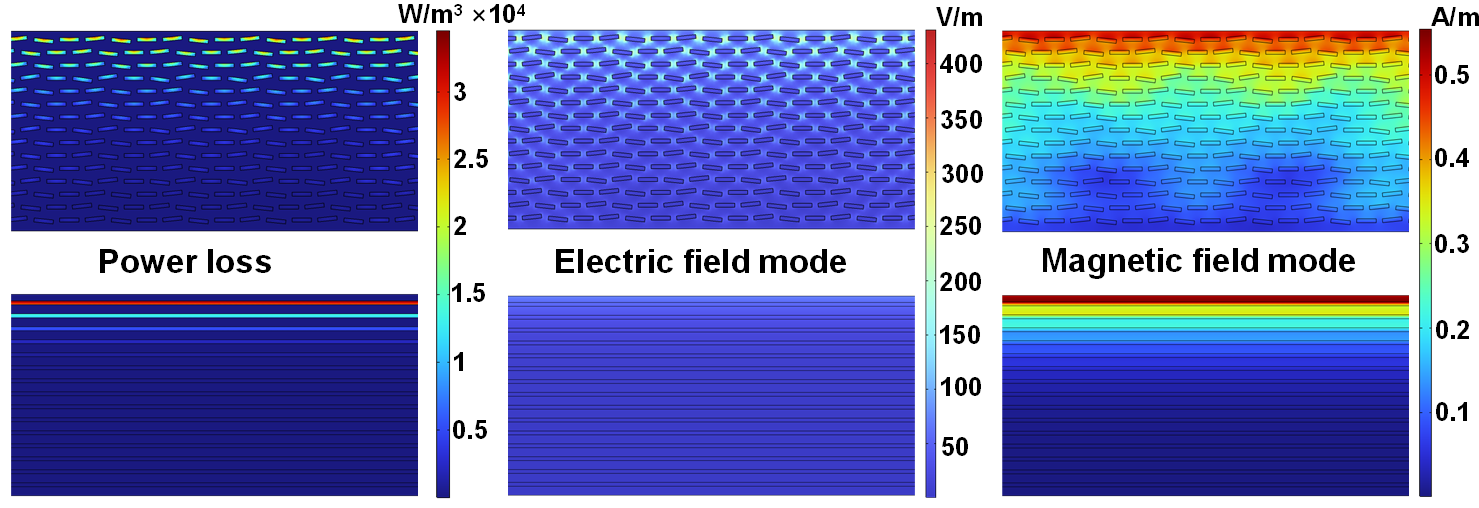


**Figure S21.** Finite element simulations for electric field mode, magnetic field mode, and total power loss distributions in AM and A@M films.


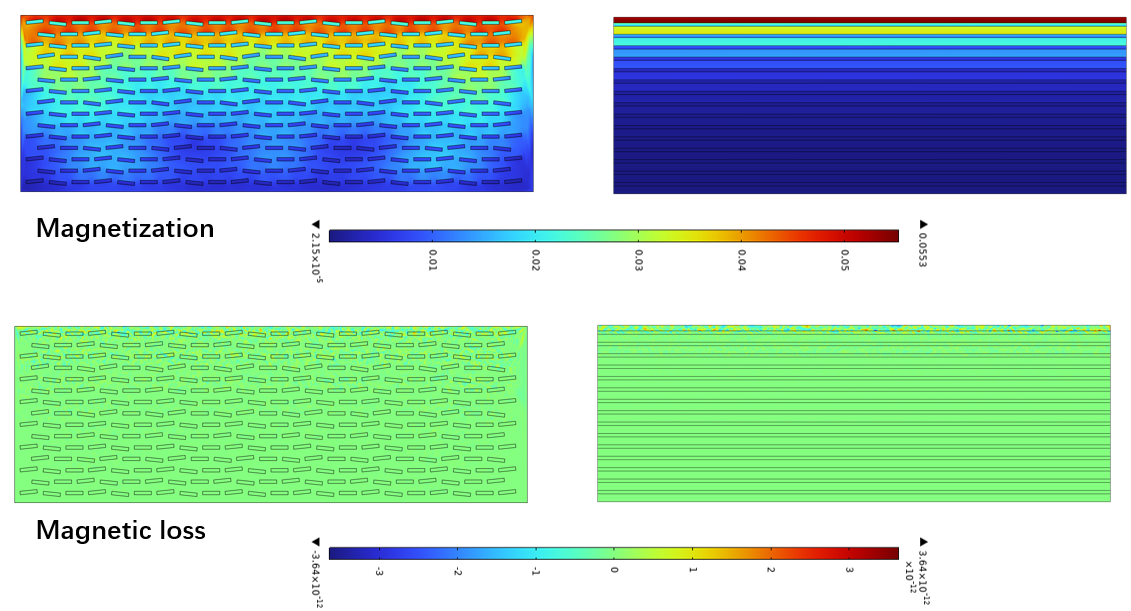


**Figure S22.** Finite element simulations for magnetization and magnetic loss in AM and A@M films.

**Figure S23.** Sheet resistance and electrical conductivity evolution of (a) A@M and (b) AM films during 80 days aging.

**Figure S24.** EMI shielding at X-band of A@M film before and after different various extreme conditions, including ultrasonic treatment, bending, acidic or alkaline environments, and extreme temperatures.

**Figure S25.** (a) Digital image of the moist heat chamber treatment process. (b) Sheet resistance and electrical conductivity evolution of A@M_5_ under high temperature and high humidity treatment for different durations, and (c) the corresponding EMI shielding efficiency curves in X band. (d) Digital image of the UV exposure treatment process (using an UV aging test chamber (LAHS-NZY), with a light source power of 4 kW.). (e) Sheet resistance and electrical conductivity evolution of A@M_5_ under UV exposure treatment for different durations, and (f) the corresponding EMI shielding efficiency curves in X band.

**Figure S26.** (a) Interpenetrating ANF/MXene layered films in various states of flexure. Relative resistance variation of the A@M_5_ film as a function of (b) repeated bending cycles (10 mm) and (c) bending radius. (d) Comparison of EMI SE before and after cyclic bending for 1000 times.

**Figure S27.** (a) Photograph, (b) FTIR spectra, (c) TEM and (d) AFM images of ANF.

**Figure S28.** (a) XRD pattern, (b) XPS spectra, (c) SEM and TEM images of MXene nanosheets

**S4. Tables**

**Table S1.** Density and MXene content of A@M and AM films.

| Samples | Density/g cm^-3^ | MXene content/wt% |
| --- | --- | --- |
| A@M_1_ | 1.036 | 10.9 |
| A@M_2_ | 1.127 | 22.7 |
| A@M_3_ | 1.199 | 33.7 |
| A@M_4_ | 1.239 | 38.3 |
| A@M_5_ | 1.248 | 41.1 |
| AM | 1.231 | 41.1 |

**Table S2.** Comparison of basic parameters of the interpenetrating ANF/MXene film with other MXene-polymer materials.

| **Samples** | **MXene Content (wt%)** | **Thickness (μm)** | **SE**  **(dB)** | **SE/t**  **(dB mm⁻¹)** | **SSE/t**  **(dB cm^2^ g^-1^)** | **Ref.** |
| --- | --- | --- | --- | --- | --- | --- |
| MXene/PVDF | 90 | 17 | 43.9 | 2523 | 19504 | [1] |
| MXene-HA | 20 | 21.6 | 43 | 1990 | 2108 | [2] |
| MXene/PVA | 19.5 | 27 | 44.4 | 1644.4 | 9343 | [3] |
| MXene/CNF | 90 | 58 | 25.8 | 444.8 | 2647 | [4] |
| MXene/CNF | 90 | 5 | 44 | 8839 | 27247 | [5] |
| MXene/PI | 50 | 41 | 57 | 1390 | -- | [6] |
| Ti3C2Tx/PEDOT:PSS | 80 | 13 | 42 | 3230 | 9169.5 | [7] |
| MXene/PU | 85 | 20 | 48 | 2400 | 13479 | [8] |
| MXene/ANF | 80 | 8 | 40.7 | 5087 | 19826 | [9] |
| MXene/PVA | 60 | 40 | 36.7 | 917.5 | 4583 | [10] |
| Ti_3_C_2_T_x_/PANI | 87.5 | 40 | 36 | 900 | -- | [11] |
| MXene/CMC-Na | 90 | 200 | 45.8 | 229 | 11595 | [12] |
| ANF-MXene-PEDOT:PSS | 30 | 36 | 45.7 | 1269 | -- | [13] |
| CNF-MXene-AgNWs | 63 | 26 | 54 | 2076 | 10543 | [14] |
| PE@PET/MXene film | ＞50 | 58 | 50.44 | 862 | 10606 | [15] |
| MXene/holocellulose nanofibrils | 64 | 35 | 40.4 | 1154 | 6444.1 | [16] |
| MXene/ the modified sawdust | 40 | 80 | 35.8 | 447.5 | 4704 | [17] |
| MXene/lignin-containing cellulose nanofibrils | 50 | 30 | 36.22 | 1207 | -- | [18] |
| MXene–pineapple leaf nanocellulose | 25 | 38 | 34 | 894.7 | 5237 | [19] |
| MXene/GEL/SL | 30 | 80 | 36 | 432 | -- | [20] |
| A@M film | 41.059 | 34.785 | 43.3 | 1244.8 | 9974.3 | This work |

**S5. References**

[1] Y. Li, B. Zhou, Y. Shen, C. He, B. Wang, C. Liu, Y. Feng, C. Shen, *Composites Part B: Engineering* **2021**, 217, 108902.

[2] J. Xiong, R. Ding, Z. Liu, H. Zheng, P. Li, Z. Chen, Q. Yan, X. Zhao, F. Xue, Q. Peng, X. He, *Chemical Engineering Journal* **2023**, 474, 145972.

[3] X. Jin, J. Wang, L. Dai, X. Liu, L. Li, Y. Yang, Y. Cao, W. Wang, H. Wu, S. Guo, *Chemical Engineering Journal* **2020**, 380, 122475.

[4] W.-T. Cao, F.-F. Chen, Y.-J. Zhu, Y.-G. Zhang, Y.-Y. Jiang, M.-G. Ma, F. Chen, *Acs Nano* **2018**, 12, 4583.

[5] Y. G. Kondarage, A. Naiduwawadu, I. Wijesinghe, C. D. H. Dharmasiri, K. L. Firestein, T. Liao, C. Yan, *Composites Part A: Applied Science and Manufacturing* **2025**, 195, 108957.

[6] L. Wang, Z. Yang, L. Lang, J. Men, T. Gao, Q. Wang, J. Cheng, Y. Liu, N. Zheng, J. Liu, X. Ji, *Advanced Composites and Hybrid Materials* **2025**, 8, 26.

[7] R. Liu, M. Miao, Y. Li, J. Zhang, S. Cao, X. Feng, *ACS Applied Materials & Interfaces* **2018**, 10, 44787.

[8] C. Huang, R. Huang, Y. Cheng, L. Zhao, N. Hu, Q. Wei, *Composites Science and Technology* **2024**, 253, 110665.

[9] C. Liu, C. Jiang, Y. Shen, B. Zhou, C. Liu, Y. Feng, *ACS Applied Materials & Interfaces* **2024**, 16, 38620.

[10] J. Y. Dong, Z. Y. Li, C. Q. Liu, B. Zhou, C. T. Liu, Y. Z. Feng, *Nano Research* **2024**, 17, 5651.

[11] Y. Zhang, L. Wang, J. Zhang, P. Song, Z. Xiao, C. Liang, H. Qiu, J. Kong, J. Gu, *Composites Science and Technology* **2019**, 183, 107833.

[12] J. Li, C. Liang, C. Lin, Y. Wang, Y. Liang, D. Dong, *Chemical Engineering Journal* **2025**, 506, 160274.

[13] S. Ma, S. Liu, J. Yan, B. Ren, H. Yu, Z. Ma, L. Sun, T. Zhang, J. Huo, Y. Yang, J. Fei, S. Chae, C. He, H. Li, *Advanced Composites and Hybrid Materials* **2025**, 8, 429.

[14] R. Cheng, B. Wang, J. Zeng, J. Li, J. Xu, W. Gao, K. Chen, *Carbon* **2023**, 202, 314.

[15] Z. Sha, H. He, H. Ma, B. Hong, J. Lu, X. Fei, M. Zhu, *Carbon* **2024**, 216, 118595.

[16] Y. He, J. Yang, W. Chen, W. Chen, L. Zhao, W. Qi, *Chemical Engineering Journal* **2023**, 464, 142565.

[17] P.-L. Wang, T. Mai, W. Zhang, M.-Y. Qi, L. Chen, Q. Liu, M.-G. Ma, *Small* **2024**, 20, 2304914.

[18] J. Zhang, S. Fu, H. Liu, B. Feng, X. Yang, B. Zhang, *International Journal of Biological Macromolecules* **2025**, 327, 147468.

[19] Y. Qian, Y. Liu, C. Wang, Y. Wei, Y. Zhang, G. Chen, J. Zhang, B. Shi, *International Journal of Biological Macromolecules* **2025**, 322, 146583.

[20] X. Zhou, X.-A. Ye, X. Zhang, D. Wen, H. Wang, G.-G. Wang, *Chemical Engineering Journal* **2025**, 503, 158401.
